# How Antisolvent-Induced Ligand Stripping Shapes CsPbX_3_ Nanocrystals and Their Assemblies

**DOI:** 10.1021/acs.nanolett.5c06380

**Published:** 2026-02-18

**Authors:** Jonas L. Hiller, Robert Thalwitzer, Ata Bozkurt, Ross Ewan Carter, Theresa Hettiger, Markus Fröhlich, Richard Hodak, Matheus Gomes Ferreira, Martin Eberle, Ekaterina Kneschaurek, Gerard N. Hinsley, Bihan Wang, Kuan Hoon Ngoi, Elke Nadler, Wojciech Roseker, Fabian Westermeier, Michael Sprung, Dmitry Baranov, Jannika Lauth, Frank Schreiber, Ivan A. Vartanyants, Marcus Scheele, Ivan A. Zaluzhnyy

**Affiliations:** † Institute of Physical and Theoretical Chemistry, 9188 University of Tübingen, Auf der Morgenstelle 18, 72076 Tübingen, Germany; ‡ Institute of Applied Physics, University of Tübingen, Auf der Morgenstelle 10, 72076 Tübingen, Germany; ¶ Division of Chemical Physics and NanoLund, Department of Chemistry, 5193 Lund University, P.O. Box 124, SE-221 00 Lund, Sweden; § 28332 Deutsches Elektronen-Synchrotron DESY, Notkestr. 85, 22607 Hamburg, Germany

**Keywords:** lead-halide perovskite, nanocrystals, supercrystals, superlattice, self-assembly, X-ray diffraction, nuclear magnetic resonance

## Abstract

Supercrystals of lead-halide perovskite nanocrystals combine the semiconducting properties of bulk perovskites with quantum confinement effects and extend them to the macroscopic scale. Supercrystals assembled via a two-layer phase diffusion process using an acetonitrile antisolvent were recently shown to be unusually robust. We investigate how the acetonitrile-assisted self-assembly process influences surface chemistry, the atomic lattice of nanocrystals, and the structure of the supercrystal. Using quantitative NMR spectroscopy, nanofocused X-ray diffraction, and optical spectroscopy, we show that a reduced density of the ligand shell caused by the exposure to acetonitrile in the assembly underlies the mechanical robustness of these supercrystals. Ligand stripping further drives a highly size-selecting lateral growth of the supercrystal and induces anisotropic relaxation of the nanocrystal atomic lattice while preserving the electronic coupling and robust light-emitting properties of the assembly. That enables the mechanical manipulation of supercrystals such as stacking, thereby opening new avenues for integration into optoelectronic devices.

## Graphical abstract

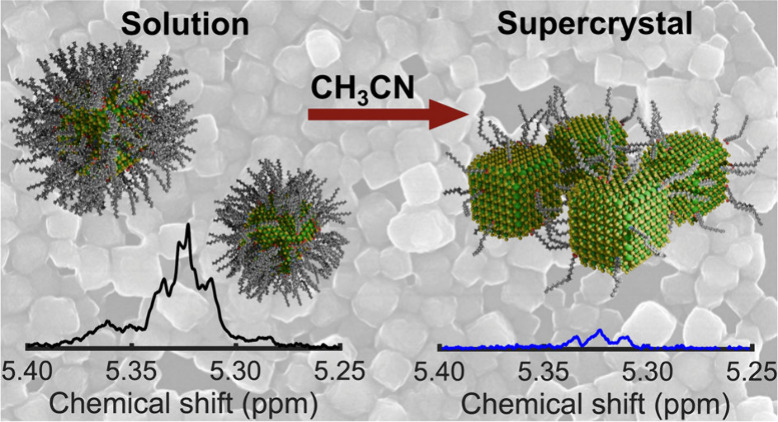

All-inorganic lead-halide perovskite nanocrystals (NCs) exhibit many attractive properties, such as high photoluminescence quantum yield (PL QY), emission energy tunability, and solution processability. Specifically, CsPbX_3_ (X = Cl, Br, I) supercrystals (SCs), highly ordered assemblies of NCs, form readily from colloidal solutions by solvent evaporation. They are macroscopic materials that retain the quantum properties of the individual NCs, rendering them an exciting research platform to study collective phenomena such as superfluorescence and superradiance.^1,2^ Beyond fundamental studies, CsPbX_3_ SCs are increasingly explored for optoelectronic device applications, such as LEDs,^3^ photodetectors,^4^ lasers,^5,6^ and superfluorescent X-ray scintillators.^7,8^

However, integrating SCs into devices by solvent-evaporation-driven self-assembly from colloidal solutions remains challenging due to limited control over the amount, size, and spatial distribution of SCs formed on the substrate. Efficient device architecture requires both the presence of a SC at the active location and the absence of residual, contaminating material elsewhere. One strategy to address these challenges is to direct SC growth using templating.^9^ Such a method, however, involves a complex multistep fabrication process that may be incompatible with specific device architectures.

In previous work, we demonstrated that by applying a two-layer (antisolvent/solvent) phase diffusion assembly, originally introduced for the assembly of metal chalcogenide semiconductor NCs,^10,11^ to the CsPbX_3_ material class, we obtained SCs displaying exceptional mechanical robustness. This stability enables the selection and precise relocation of SCs from their growth substrates to arbitrary target locations using microgrippers, thereby addressing both requirements for device integration.^12^

In the present work, using quantitative nuclear magnetic resonance (NMR) spectroscopy, we demonstrate that exposure to acetonitrile during the assembly invokes a substantial loss of surface-bound ligands. If these robust SCs are to be used in optoelectronic devices, it is essential to understand how this ligand loss affects the structural and optical properties of the NCs in the assemblies. Here, we address these questions by employing a combination of X-ray scattering with a nanofocused beam^13^ and diffraction-limited optical spectroscopy. Structural analysis reveals anticorrelated radial gradients of the in-plane atomic lattice parameters, which we attribute to lattice relaxation following ligand removal. Complementary spectroscopic analysis reveals that the assembled SCs remain robust light emitters, exhibiting enhanced emission red-shifts arising from efficient electronic coupling due to the reduced ligand shell. We showcase that SCs treated in this way enable the fabrication of new device architectures consisting of several CsPbX_3_ SCs with different halide compositions. The combination of such distinctly different SCs would be difficult to realize by co-crystallization from solution due to the rapid halide ion exchange in CsPbX_3_.^14^

Figure 1a shows photographs of the assembly process under ambient light (top) and UV illumination (lower). In an inert atmosphere, a substrate, such as a Si wafer, is placed in a test tube, and acetonitrile (CH_3_CN) is added until the substrate is approximately halfway covered. The acetonitrile phase is overlayered with the colloidal solution of CsPbX_3_ NCs in hexane. The test tube is placed inside a falcon tube, which is sealed and left at room temperature for 5 days under exclusion of light.

**Figure 1:**
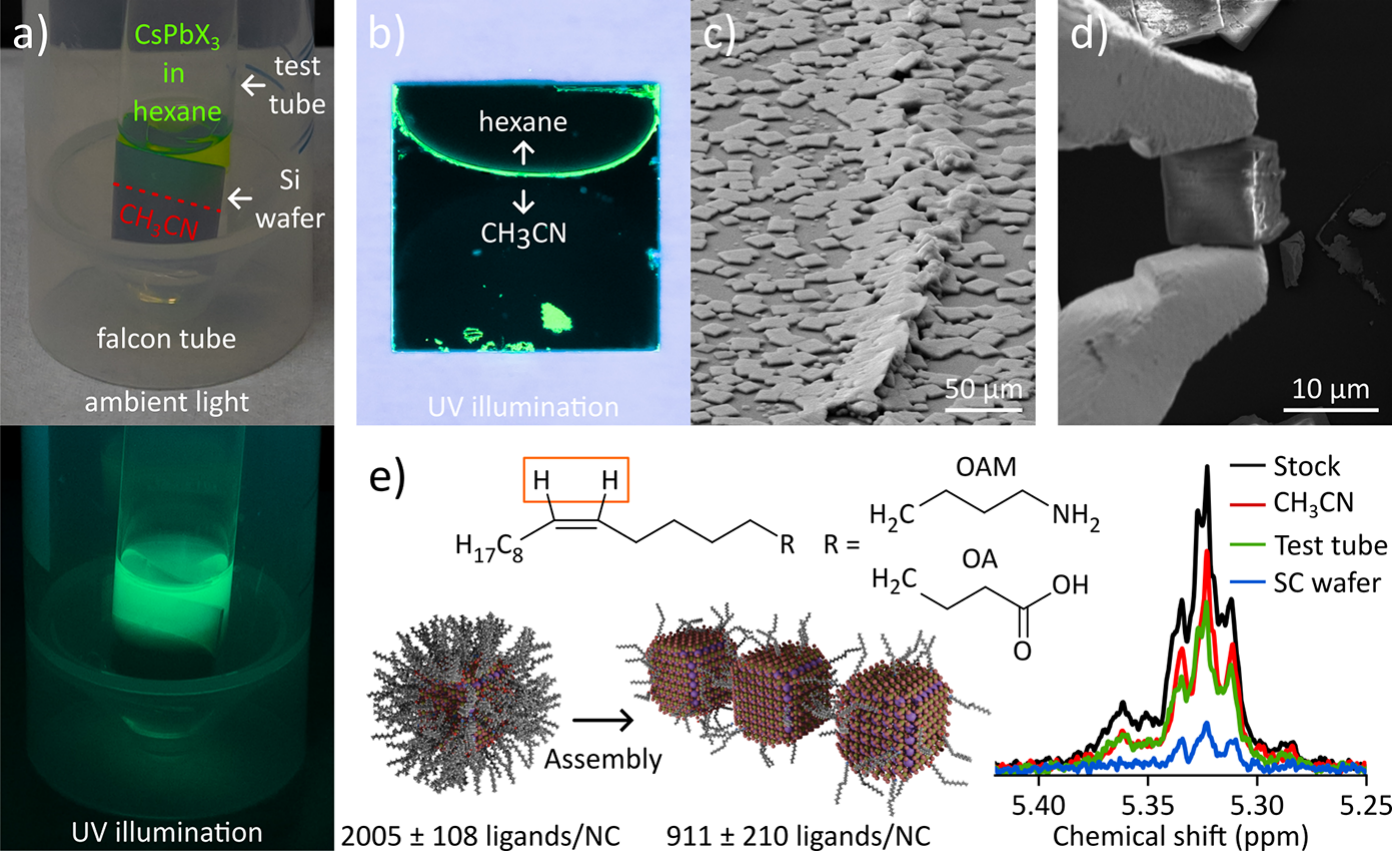
a) Photograph of the SC assembly process under ambient light (top) and under UV illumination (lower). b) Distribution of SCs on the Si wafer obtained after assembly. c) Scanning electron microscopy (SEM) micrograph of the pile-up of SCs at the former phase boundary. d) SEM micrograph of a CsPbBr_3_ SC picked up using a microgripper. e) Sketch of ligand loss during the assembly process determined by quantitative ^1^H NMR spectra of the vinyl signals of oleylamine (OAM) and oleic acid (OA).

After 5 days, the hexane phase has evaporated, and the substrate is removed from the test tube. The vast majority of CsPbBr_3_ is deposited as SCs on the substrate close to the former phase boundary as can be seen in the photograph of the UV illuminated substrate in Figure 1b. A fluorescent, curved line spanning the entire length of the phase boundary is observed with the convex of the line consistently pointing toward the acetonitrile phase. As can be seen in the closeup SEM image of the line in Figure 1c, the very center of the line consists of a pile-up of supercrystals, which is situated between numerous isolated SCs with lateral sizes ranging from 5 × 5 μm^2^ to 25 × 25 μm^2^. Typically, the width of the crystallization line, consisting of the central pile-up and the surrounding SCs, is on the order of 1 mm. By choosing the position of the phase boundary on the substrate in the assembly, we have rudimentary control over where SCs form on the substrate. The exceptional mechanical hardness of SCs obtained from this assembly allows for their fine positional control using microgrippers, as shown in the SEM micrograph in Figure 1d. In our previous study, we speculated that the approximately 10-fold increase in the hardness of the SC exposed to acetonitrile could result from a decreased density of the soft ligand shell surrounding the NCs.^12^ By employing quantitative NMR we can now provide evidence for this hypothesis, as shown in Figure 1e.

The NCs are capped with a mixture of oleylamine (OAM) and oleic acid (OA). Quantification of surface ligands was performed using the ^1^H NMR signal of the vinyl protons (highlighted by the frame in Figure 1e). Although the ^1^H vinyl signals of the OAM and OA overlap and cannot be distinguished, the combined vinyl signal allows quantification of the total number of ligands. For quantitative analysis, ethylene carbonate, dissolved in deuterated dimethyl sulfoxide (DMSO-*d*_6_), was used as an external standard. This solution was added to several samples: the stock solution used for the self-assembly (black curve), the residual acetonitrile remaining in the test tube after the assembly (red curve), the material adhered to the inner wall of the test tube (green curve), and finally, the DMSO solution was used to wash the SCs from the crystallization substrate (blue curve). DMSO fully decomposes the NCs, releasing all surface-bound ligands and enabling quantification of the free ligands.

The ^1^H NMR spectra displayed in Figure 1e are normalized relative to the external standard, allowing for a direct comparison between the samples. Based on the integrated vinyl proton signals, the total number of ligands were determined. After normalization to the number of ligands initially present in the stock solution, 51% of the ligands were found in the residual acetonitrile, 38% were recovered from the inner wall of the test tube, and 11% were found on the SC substrate. These results demonstrate that ligands are present in the residual acetonitrile where no NCs are observed. This suggests that either (i) the stock solution contains a significant number of free ligands, (ii) the majority of NCs decompose during the assembly, or (iii) ligands are stripped from the NC surfaces by the acetonitrile antisolvent.

The concentration of NCs in the stock solution was determined by UV/vis-spectroscopy (Figure S1 in the Supporting Information (SI)).^15^ From the known volume of stock solution used in both the assembly and the NMR quantification, the total amount of NCs was calculated to be 4·10^–10^ mol. Based on this value, we can calculate the average number of ligands per NC in the stock solution and give an estimate for the value on the substrate after the assembly. For CsPbBr_3_ the theoretical density of bound surface ligands is reported to be 2.9 ligands/nm^2^.^16^ Assuming cubic NCs with an average edge length of 10.8 nm in the stock solution (Figure S2 in the SI), this corresponds to an expected value of 2029 ligands/NC. Based on our measured total ligand content and the calculated amount of NCs, we determine the value of 2005 ± 108 ligands/NC in the stock solution, an excellent agreement that indicates that only a few if any unbound ligands are expected to be present in the stock solution. Consequently, the 51% of ligands found in the acetonitrile after the assembly cannot be explained by excess free ligands. While NCs, unlike ligands, are not necessarily conserved, we observe overall NC stability against acetonitrile and thus rule out the degradation of a significant fraction of the NCs. We therefore conclude that the vast majority of the 51% of ligands found in the residual acetonitrile were stripped from NC surfaces by the antisolvent during the assembly.

The number of ligands per NC on the crystallization substrate cannot be directly determined but can be estimated under two assumptions. First, we assume that the number of NCs remains constant during the assembly process. Second, the fraction of NCs crystallizing on the substrate versus those adhering to the inner wall of the test tube following assembly must be determined. From optical absorbance spectroscopy of NC dispersions obtained from the crystallization experiments, we determine that on average 27 ± 6% of the NC material is deposited on the substrate (see Section 1.6 in the SI). The corresponding ligand coverage is calculated to be 911 ± 210 ligands/NC for the NCs incorporated into the SCs on the wafer. These results show that the assembly using acetonitrile drastically reduces the average number of surface-bound ligands on the NCs from a full surface coverage of 2005 ± 108 ligands/NC in the stock solution to around 911 ± 210 ligands/NC within a SC. To investigate how the loss of ligands affects the structure of NCs in the assembled superstructures, we performed X-ray nanodiffraction experiments, focusing on the wide-angle X-ray scattering (WAXS) regime. The complementary analysis of the SCs based on small-angle X-ray scattering (SAXS) is reported in our previous work.^12^ Figure 2a shows the spatially averaged diffraction pattern of the entire CsPbBr_3_ SC. The diffraction peaks at small angles arise from the periodic arrangements of NCs in the SCs. At larger scattering angles, three Bragg peaks, corresponding to atomic lattice reflections, are recorded and indexed in pseudocubic notation as 100_AL_, 110_AL_, and 200_AL_.^12,17^

**Figure 2:**
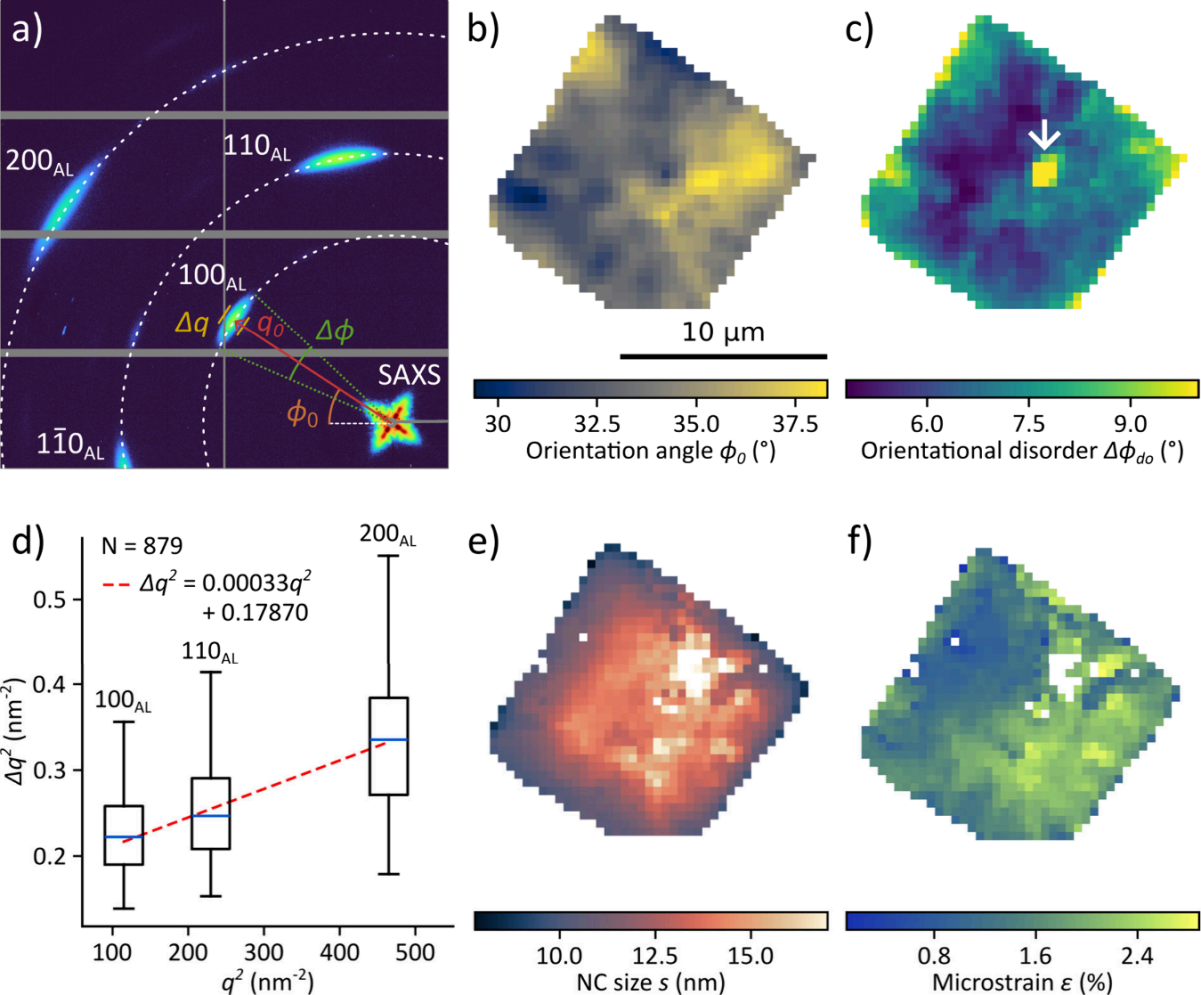
Results of WAXS X-ray nanodiffraction experiments performed on a CsPbBr_3_ SC obtained from the two-layer phase diffusion assembly using acetonitrile as antisolvent. a) Spatially averaged diffraction pattern of the SC with structural parameters of interest indicated for the 100_AL_ Bragg peak. b) Map of the in-plane orientation angle ϕ_0_ determining the in-plane orientation of the atomic lattice with respect to the horizontal axis, obtained for the 100_AL_ reflection. c) Map of the orientational disorder *Δϕ*_*do*_, calculated from the azimuthal width of the 100_AL_ reflection, corrected for NC size Scherrer broadening and strain. The white arrow points to an area of high orientational disorder likely caused by pressing on the SC with a microneedle. d) Williamson-Hall (WH) analysis of the three atomic lattice reflections for each pixel of the SC. The dashed red line shows the linear fit of the median values. e) Map of the NC size *s* derived from the WH fit intercept at each pixel. f) Map of the microstrain *ε* calculated from the WH fit slope at each pixel. Missing pixels in e) and f) correspond to diffraction patterns for which WH analysis yielded unphysical results.

The diffraction patterns are transformed into polar coordinates, and the three atomic lattice Bragg peaks are fitted using 2D-Gaussians. Besides the overall amplitude, fitting yields four parameters: the radial peak position *q*_0_, the radial peak width Δ*q*, the azimuthal peak position ϕ_0_, and the azimuthal peak width Δϕ. Among these, ϕ_0_ is mapped directly to visualize the local orientation of the atomic lattice, while the other parameters are used to calculate lattice spacing and strain (from *q*_0_), nanocrystal size and microstrain (from *q*_0_ and Δ*q*), and orientational disorder (from Δϕ after correction for size and strain contributions). These structural quantities are discussed in the following.

Figure 2b shows the average in-plane orientation angle of the atomic lattice, extracted from the 100_AL_ reflection. The map reveals a clear in-plane crystallographic axis, with deviations of about ± 5° from the mean orientation. These deviations are not random but appear as extended domains spanning multiple pixels with a shared preferential orientation.

Figure 2c maps the azimuthal broadening of the 100_AL_ reflection corrected for size and strain contributions. The resulting parameter Δϕ_do_ quantifies the degree of orientational disorder of the atomic lattices in the illuminated volume. The SC center, likely the nucleation point, shows a relatively low level of disorder. As crystal growth proceeds outward, a gradual loss of alignment occurs, with respect to the SC center, as indicated by an increasing azimuthal broadening. The cluster of crystals close to the SC center (indicated by the white arrow), which strongly deviate from this trend, showing the highest disorder, is likely a result of the microneedle used to position the crystal on the Kapton substrate.

Williamson-Hall (WH) analysis was performed to separate the radial peak broadening into contributions from finite nanocrystal size *s* and fluctuations of lattice distortions *ε* (microstrain). Figure 2d shows the distributions of squared radial broadening Δ*q* ^2^ as a function of *q* ^2^ for the three atomic lattice peaks across all 879 measured positions of the SC and a linear fit Δ*q* ^2^ = *ε* ^2^·*q* ^2^ + Δ*q*_0_^2^.^17–19^ The broadening is not uniform but varies substantially throughout the sample. From the linear fit, we obtained the average microstrain *ε* and the NC size as *s* = 2*πK*/Δ*q*_0_ where *K* = 0.85 is the average shape factor for cubic NCs.^20^ The average NC size is *s* = 12.6 nm and the average microstrain is *ε* = 1.8%. Spatially resolved maps of these parameters, displayed in Figure 2e,f, are obtained by performing WH analysis independently at each position on the SC.

The NC size map in Figure 2e reveals a pronounced gradient, ranging from 8.5 nm at the SC edges to 16 nm in the SC center, matching both qualitatively and quantitatively the SC lattice constant gradient we previously reported from SAXS measurements on this sample.^12^ Combined with the orientational disorder map, this size gradient supports a model in which nucleation during the two-layer diffusion assembly is initiated by larger NCs. The microstrain map in Figure 2f quantifies local fluctuations of lattice strain across the SC. Unlike the NC size, the microstrain does not follow a center-to-edge gradient but instead appears patchy, with domain-like regions of higher strain interspersed with lower strain areas, indicating that local packing frustration and defect accumulation are the main contributors to fluctuations of lattice strain. The average microstrain of 1.8% is significantly higher than typical values for bulk crystals (<0.1%)^21,22^ but is entirely consistent with nanocrystal assemblies, where large surface-to-volume ratio, ligand interactions, and packing mismatch often generate microstrain of 1–5%.^17,23^

From the *q*_0_-values of the 100_AL_ and 110_AL_ peak, we calculated the pseudocubic in-plane atomic lattice parameters *a* and *b*. The maps of the lattice parameters *a* and *b* across the SC in Figure 3a,b exhibit systemic variations along the direction of the radial NC size gradient shown in Figure 2e. The lattice parameter *a*, extracted from *q*_0_(100_*AL*_), decreases toward the center of the SC, where the largest NCs are located. The lattice parameter *b*, calculated from the radial positions of both *q*_0_(100_*AL*_) and *q*_0_(110_*AL*_), exhibits the opposite trend, increasing toward the center. While subtle, the variations are larger than the instrumental resolution, and the underlying changes in the radial positions of the atomic lattice reflections are even discernible by eye in the raw diffraction patterns recorded from the edges and the center of the SC (Figure S9 in the SI).

**Figure 3:**
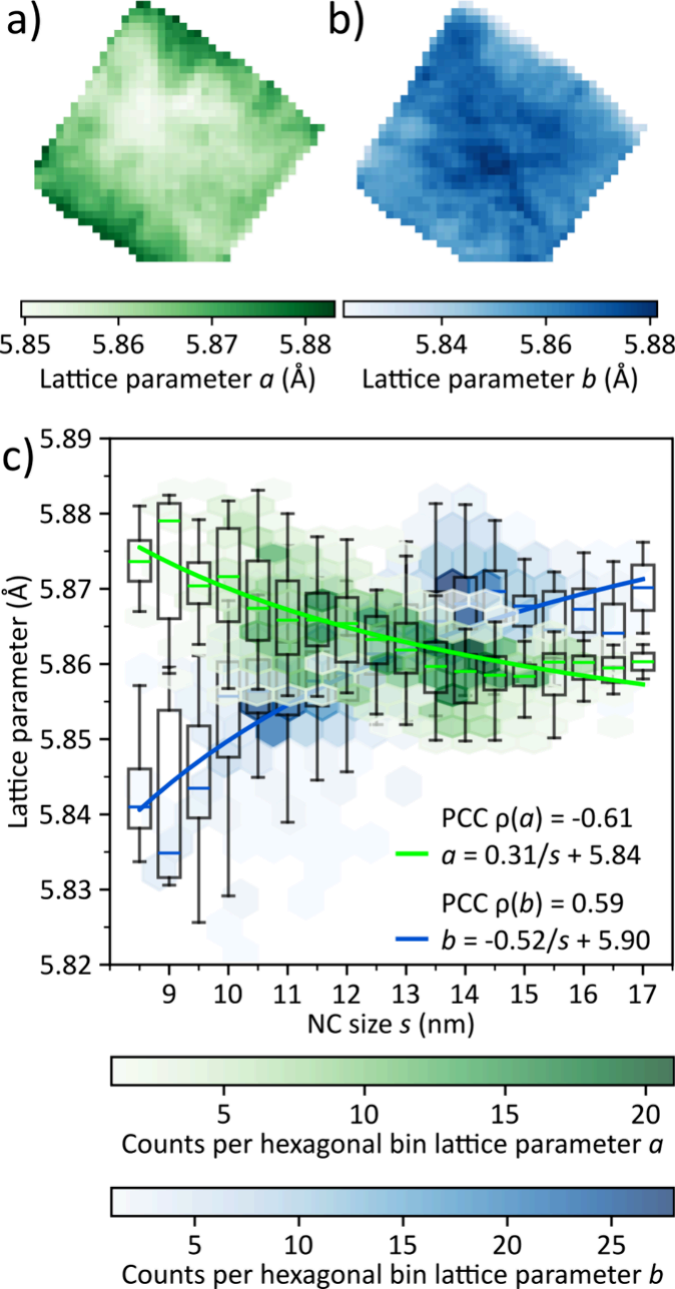
In-plane atomic lattice parameters of the CsPbBr_3_ SC are from Figure 2a. Map of the pseudocubic lattice parameter *a*. b) Map of the pseudocubic lattice parameter *b*. c) Correlation of lattice parameters *a* and *b* with NC size. Hexagonal binning displays the statistical distribution, while boxplots indicate the spread and median value within each 0.5 nm size interval. The Pearson correlation coefficients (PCC) ρ are stated to quantify the degree of linear correlation. The median values of the boxplots are fitted using an empirical power-law relation: *a* – *a*_0_ ∝ *s* ^–α^.^24,25^

To quantify these correlations, Figure 3c plots lattice parameters *a* and *b* as functions of the NC size. A pronounced anticorrelation with NC size is observed for *a* (ρ = −0.61), whereas *b* displays a strong positive correlation (ρ = 0.59). The difference between the lattice parameters is largest for small nanocrystals (*a* – *b* = 0.035 at *s* = 8.5 nm), vanishes at around 13 nm size and then reemerges less strongly for larger NCs (*b* – *a* = 0.015 at *s* = 17 nm). We see the same trend in the in-plane lattice parameters for SCs of different halide compositions, namely, CsPbBr_2_Cl and CsPbCl_3_ (Figures S10–S11 in the SI).

The X-ray nanodiffraction experiments discussed so far were performed on flat-lying crystals with transmission geometry. In this configuration, only reflections with a Miller index *l* = 0 are accessible, meaning that the out-of-plane lattice parameter *c* cannot be probed. To access this parameter, we took advantage of the mechanical robustness of the antisolvent-grown SCs, used microgrippers to rotate a SC and position it upright on a Kapton substrate. The nanodiffraction measurements performed on this rotated SC are displayed in Figure 4.

**Figure 4:**
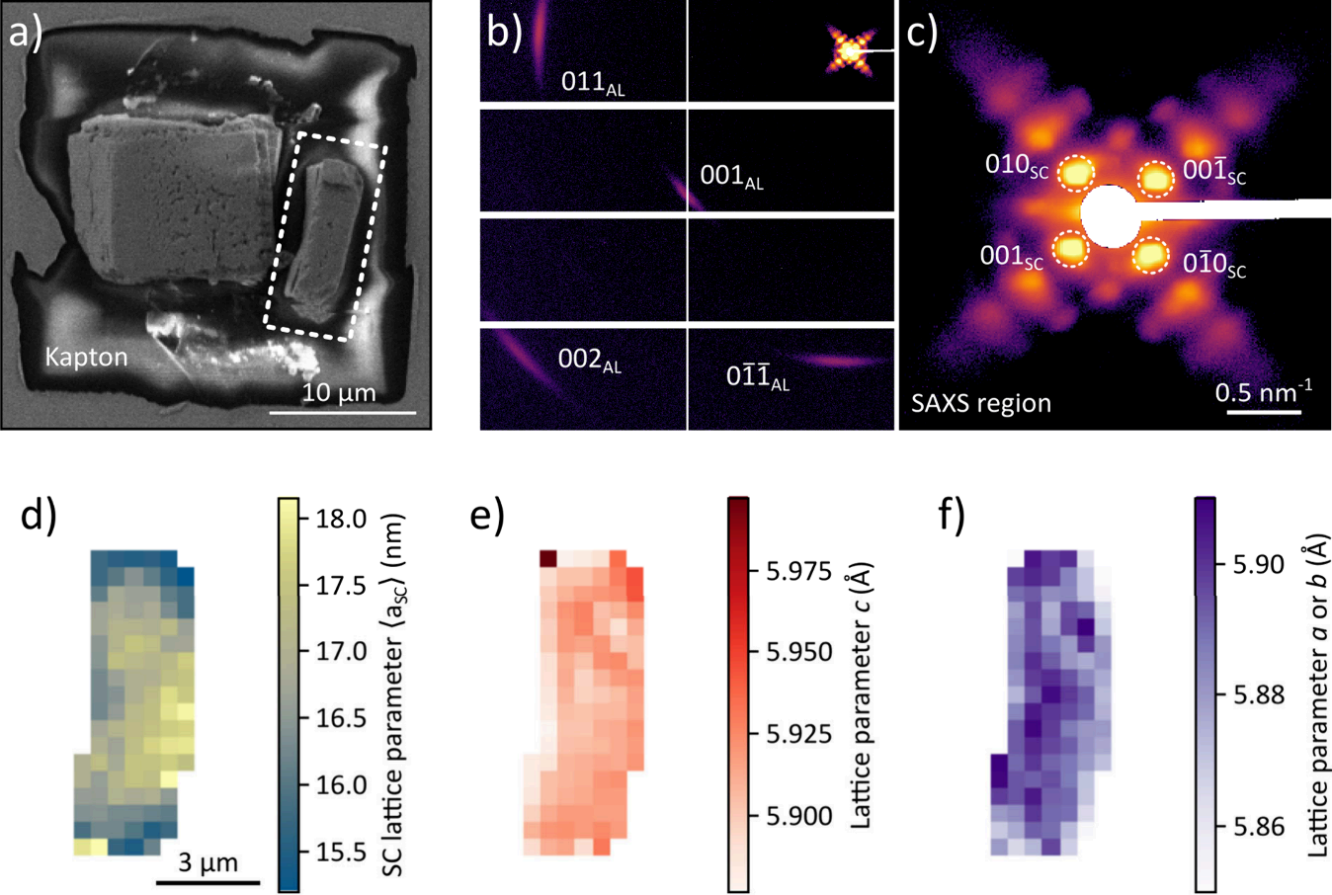
Results of X-ray nanodiffraction experiments performed on a CsPbBr_3_ SC obtained from the two-layer phase diffusion assembly using acetonitrile as antisolvent. a) SEM micrograph of the sample consisting of a flat-lying (left) and an upright-standing (right) SC on a Kapton window. The analysis focuses on the upright SC, highlighted by the dashed white lines. b) Spatially averaged diffraction pattern recorded from the upright SC, with a closeup of the SAXS region in c). d) Map of the average SC lattice parameter *⟨*a_SC_ *⟩*. e) Map of the out-of-plane lattice parameter *c*. f) Map of the ambiguous in-plane lattice parameter *a* or *b*.

The patterns recorded from the upright CsPbBr_3_ SC highlighted by the white dashed lines in Figure 4a were used for nanodiffraction analysis. Figure 4b displays the spatially averaged diffraction pattern of the entire SC. From the *q*_0_-values of the four first-order SAXS peaks, indicated in the close-up view of the spatially averaged SAXS region Figure 4c, we extract the two SC lattice parameters *a*_1_ and *a*_2_. From these, we calculate the average SC lattice parameter ⟨*a*_SC_⟩ = (*a*_1_ + *a*_2_)/2. This parameter represents the average center-to-center distance of NCs in the SC, accounting for both the NC size and interparticle spacing. The map of ⟨*a*_SC_⟩ in Figure 4d reveals an increase of the SC lattice parameter toward the SC center, in accordance with the NC size increase observed in the flat-lying SCs. Notably, the increase in ⟨*a*_SC_⟩ is limited to the in-plane direction with respect to a flat-lying SC. This agrees with previous SEM results, where no significant NC size gradient was observed along the out-of-plane direction of a cleaved SC surface.^12^

In this geometry, the out-of-plane lattice parameter *c* can be directly calculated from *q*_0_(001_*AL*_) and is mapped in Figure 4e. The results show that c, with a median value of 5.910 Å, is slightly larger than *a* and *b* and exhibits no clear radial trend. From the *q*_0_-values of the 001_AL_ and 011_AL_ peaks, one of the in-plane lattice parameters is calculated, however, it cannot be unambiguously assigned to be *a* or *b*.

At room temperature, bulk CsPbBr_3_ crystallizes in the orthorhombic phase, but the structure of CsPbBr_3_ NCs remains an active and open discussion. Brennan et al. looked at 5 and 10 nm CsPbBr_3_ NCs at the single-particle level via a high-resolution transmission electron microscopy (HRTEM) defocus-series analysis. They found that CsPbBr_3_ NC lattices are exclusively cubic for *l* ∼ 5 nm NCs and predominantly cubic but with a minority orthorhombic phase for 10 nm NCs, suggesting the presence of a size-dependent change in CsPbBr_3_ NC crystal symmetry.^26^ This is consistent with other reports of phase coexistence in single CsPbBr_3_ nanoplatelets and nanocrystals.^27,28^ In our nanodiffraction experiments, each pixel represents the ensemble diffraction signal of many NCs within an illuminated volume of approximately 300 nm × 300 nm × 3 μm (crystal thickness). Consequently, no conclusions about phase coexistence at the NC level can be drawn.

Beyond size, the phase has also been linked to the synthesis conditions and surface chemistry. Protesescu et al. suggested that synthesis temperatures above 130 °C, together with the presence of surface passivating ligands, stabilize CsPbBr_3_ NCs in their high-temperature cubic phase.^29^ The NC synthesis employed in the work involved a hot injection at above 200 °C, yielding NCs with a full ligand surface coverage (Figure 1). Following the findings of Protesescu et al., it is highly likely that these conditions lead to stabilization of the thermodynamically unfavorable cubic phase. This is consistent with X-ray diffraction measurements of the NCs in reflection geometry prior to exposure to acetonitrile (Figure S12 in the SI). During the assembly, surface ligands are removed, and the nanocrystal lattice relaxes to minimize surface energy. Our data indicate that this relaxation of the in-plane lattice parameters is at least partly anisotropic. NCs of different sizes appear to relax to different extents. Since smaller NCs have a higher surface-to-volume ratio, it is reasonable that relaxation induced by ligand removal produces a stronger response there. Independently of that, a greater in-plane anisotropy for smaller nanocrystals is consistent with a report by Bertolotti et al.^30^ As SCs often adhere to the microgrippers in the transfer (Figure 5a), we use a micromanipulator needle to slip off the SC for accurate positioning (Figure 5b,c). We believe this pressing down with the needle on the SC to be the cause of the locally strongly increased orientational disorder in the center of the SC displayed in Figure 2e. An example of a CsPbBr_2_Cl SC stacked on top of a CsPbBr_3_ SC is displayed in Figure 5d-f. Such a heterostructure would be challenging to obtain by co-crystallization from solutions due to the fast halide ion exchange in CsPbX_3_.

As displayed in Figure 5, the mechanical hardness of the SCs assembled via the antisolvent approach allows for selecting individual SCs and transferring them between substrates in an SEM. This is typically possible with only minimal deformation of the SCs.

**Figure 5:**
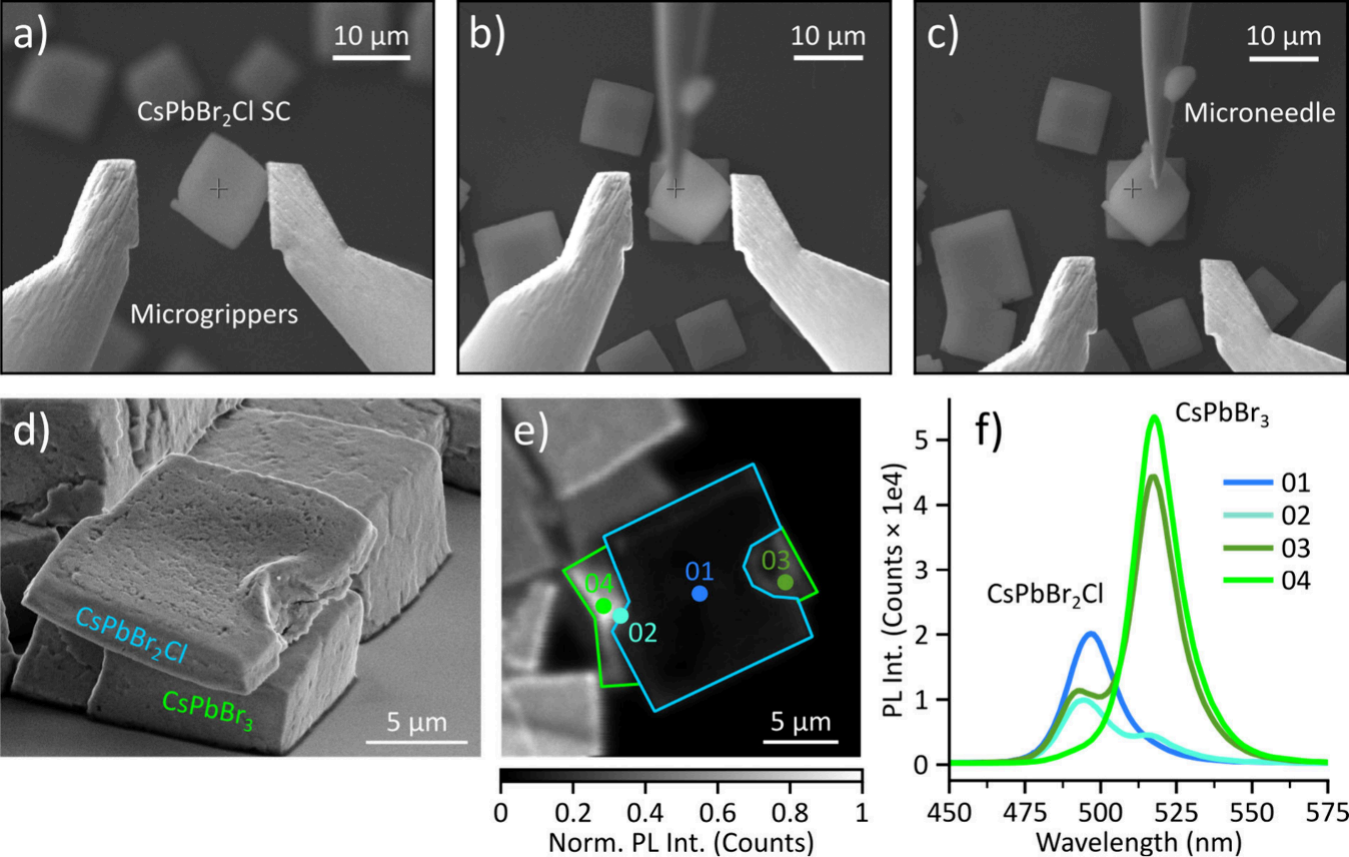
a) SEM image of a CsPbBr_2_Cl SC that adheres to the microgrippers after lifting from the growth substrate. b) The SC is transferred to a location of choice, here on top of another SC. A micromanipulator needle is lowered onto the SC. c) The micromanipulator needle holds the SC in place, allowing the grippers to be removed. d) SEM image of a CsPbBr_2_Cl SC stacked on top of a CsPbBr_3_ SC. e) Confocal PL image of the stacked SCs. f) Spatially resolved emission spectra, recorded from the positions indicated in e).

The influence of ligand stripping during the two-layer phase diffusion assembly process on the optical properties of CsPbBr_3_ NCs and the obtained superstructures was investigated by using steady-state emission (Figure S13 in the SI), femtosecond transient absorption spectroscopy (Figure S14 in the SI), and confocal photoluminescence (PL) and lifetime mapping (Figures S15–S18 in the SI). A key finding is that upon stirring a solution of CsPbBr_3_ NCs in hexane with acetonitrile, the photoluminescence quantum yield (PLQY) drops from 90% to 50% within the first 24 h, after which it stays constant for at least 5 days. This is consistent with literature reports that perovskite NCs can remain robust light emitters even after substantial ligand loss from their surfaces.^31,32^ Spatially resolved spectroscopy reveals radial gradients in emission energy, fluorescence lifetime, and spectral line shape throughout the SCs, which we attribute to result from the presence of NC size and orientational disorder gradients.

In conclusion, we provide evidence for a pronounced loss of ligands of CsPbX_3_ NCs during the two-layer phase diffusion assembly using an acetonitrile antisolvent. This reduction of the soft, interdigitated ligand sphere explains the previously reported mechanical robustness of SCs obtained from this assembly.

We performed X-ray nanodiffraction experiments on SCs with different orientations. Maps of NC size-distribution, microstrain, and orientational disorder reveal that SC growth likely initiates from large NCs in the SC center, incorporating progressively smaller NCs as it grows radially, leading to an increase in orientational disorder. A possible explanation lies in the concept of colloidal softness: as NC size increases, the ratio of ligand length to NC size decreases, making the packing in the SC center mechanically stiffer, whereas the outer region, composed of smaller NCs, remains “softer” and more easily deformable.^33^ We propose that the resulting accumulation of orientational disorder may impose a limit on the lateral dimensions of the SCs obtained from this assembly method.

The spatially resolved SC lattice parameters extracted from an upright SC indicate that the NC size gradient is limited to the radial direction. Accompanying the radial NC size gradient, we observe anticorrelated radial gradients of the in-plane atomic lattice parameters *a* and *b*, while no clear gradient is detected in the out-of-plane direction. We attribute these changes to the NC lattice relaxation following ligand removal during the assembly process.

From optical measurements, we find that although ligand removal adversely affects the PL quantum yield, the SCs remain robust light emitters. In this context, the two-layer phase diffusion assembly offers substantial flexibility, as parameters such as the amount and type of antisolvent, the employed NC concentration, and the ligand composition can be tuned to potentially improve the balance between mechanical robustness and optical performance. Another promising strategy to improve the PLQY is postassembly treatment of the SCs, for example by the addition of PbBr_2_ ^34^ or oleylamine ligand reintroduction.^16^ Spatially resolved spectroscopy reveals radial gradients in emission energy, fluorescence lifetime, and spectral line shape throughout the SC, which are primarily attributed to the corresponding radial gradient in the size distribution (see Figure S15 and accompanying discussion in the SI). Although dominated by NC size in this case, the spectral shifts in the SCs have multiple origins, including strain, as reported by Lapkin et al.^17^ and suggested by Levy et al.^35^ , for SCs with higher size uniformity. Another potential contribution stems from the electronic coupling of the NCs in the assembly. Reducing the number of surface bound ligands is likely to allow for more interdigitation of the ligand spheres, leading to a smaller equilibrium interparticle distance and increased coupling of the NCs in the SC.^36,37^

The mechanical robustness of the CsPbX_3_ supercrystals obtained by the acetonitrile-assisted assembly process opens new avenues for their integration into optoelectronic and photonic devices. For certain applications, the observed reduced density of the insulating ligand may be beneficial, for instance by facilitating charge injection in perovskite LEDs.^38^

## Supplementary Material



## Notes

Details on materials used for the synthesis of perovskite NCs and assembly of the SCs. Description of the experimental techniques: NMR, steady-state optical measurements, spatially resolved optical measurements, time-correlated single photon counting, transient absorption spectroscopy, electron microscopy, and spatially resolved X-ray nanodiffraction. Characterization of the perovskite NCs in stock solution, details of quantitative NMR analysis of oleyamine and oleic acid. Characterization of SCs. Details on the analysis of X-ray diffraction data collected at synchrotron and laboratory sources. Results of the optical measurements: quantum yield, transient absorption, photoluminescence, fluorescence, and time-correlated single photon counting (PDF)
