# Supplementary material for: How Antisolvent-Induced Ligand Stripping Shapes CsPbX3 Nanocrystals and Their Assemblies
Source: Nano Lett. 2026 Feb 18;26(8):2955–63. doi: 10.1021/acs.nanolett.5c06380 (PMC12964548; doi:10.1021/acs.nanolett.5c06380)
Supplement: Supplementary file 1 [file nl5c06380_si_001.pdf]

Supporting Information for

How antisolvent-induced ligand stripping shapes $CsPbX_3$ nanocrystals and their assemblies

Jonas L. Hiller,[1] Robert Thalwitzer,[1] Ata Bozkurt,[1] Ross Ewan Carter,[2] Theresa Hettiger,[1] Markus Fröhlich,[1] Richard Hodak,[1] Matheus Gomes Ferreira,[3] Martin Eberle,[1] Ekaterina Kneschaurek,[2] Gerard N. Hinsley,[4] Bihan Wang,[4] Kuan Hoon Ngoi,[4] Elke Nadler,[1] Wojciech Roseker,[4] Fabian Westermeier,[4] Michael Sprung,[4] Dmitry Baranov,[3] Jannika Lauth,[1] Frank Schreiber,[2] Ivan A. Vartanyants,[4] Marcus Scheele[1]* and Ivan A. Zaluzhnyy[2]*

[1] Institute for Physical and Theoretical Chemistry, University of Tübingen, Auf der Morgenstelle 18, 72076 Tübingen, Germany

[2] Institute of Applied Physics, University of Tübingen, Auf der Morgenstelle 10, 72076 Tübingen, Germany

[3] Division of Chemical Physics and NanoLund, Department of Chemistry, Lund University, P.O. Box 124, SE-221 00 Lund, Sweden

[4] Deutsches Elektronen-Synchrotron DESY, Notkestr. 85, 22607 Hamburg, Germany

*Corresponding authors

1. Properties of the $CsPbBr_3$ NCs **2**

1.1 Chemicals **2**

1.2 Synthesis of $CsPbX_3$ nanocrystals and assembly of SCs **2**

1.3 $CsPbBr_3$ stock solution concentration **2**

1.4 $CsPbBr_3$ NC stock solution size distribution **4**

1.5 Qualitative NMR of oleylamine and oleic acid **4**

1.6 Quantitative NMR **5**

1.7 Influence of acetonitrile exposure on NC size distribution **8**

1.8 Further information on NC self-assembly **9**

2. X-ray nanodiffraction in transmission geometry **12**

2.1 Separation of contributions to azimuthal broadening **12**

2.2 Calculations of the in-plane atomic lattice parameters **13**

2.3 Calculation of the average SC lattice parameter **13**

2.4 Instrumental resolution **13**

2.5 Spatially-resolved in-plane atomic lattice parameters of a $CsPbBr_2Cl$ and a $CsPbCl_3$ SCs **15**

3. X-ray characterization of as-synthesized $CsPbBr_3$ nanocrystals **17**

4. Additional Spectroscopy Data **19**

4.1 Quantum yields **19**

4.2 TA Spectroscopy **20**

4.3 Spatially Resolved PL Spectroscopy **21**

4.5 Fluorescence lineshape analysis **23**

4.6 Time-Correlated Single Photon Counting **25**

1. Properties of the $CsPbBr_3$ NCs

1.1 Chemicals

1-Octadecene (ODE), technical grade, 90%, Sigma Aldrich; Oleic acid (OA), 97%, Acros Organics; Oleylamine (OAM), 80-90%, Acros Organics; Cesium carbonate (Cs_2CO_3), 99.99% (trace metal basis), Acros Organics; Lead(II)chloride ($PbCl_2$), 99.999% (trace metal basis), Sigma Aldrich; Lead(II)bromide ($PbBr_2$), 99%, Acros Organics; Lead(II) acetate trihydrate (PbOAc), Sigma Aldrich, 99.99% (trace metal basis); n-hexane, 97% extra dry over molecular sieve, AcroSeal, Acros; Acetonitrile, 99.9% extra dry over molecular sieve, AcroSeal, Acros. All chemicals were used as purchased.

1.2 Synthesis of $CsPbX_3$ nanocrystals and assembly of SCs

$CsPbX_3$ NCs were synthesized using a slightly modified hot-injection method published by Dutta et al.[1] Therefore, 97 mg (0.3 mmol) Cs_2CO_3 and 227 mg (0.6 mmol) PbOAc were loaded into a 50 mL three neck flask and dissolved in 30 mL ODE and 3 mL oleic acid. The mixture was degassed under vacuum at 120 °C for 2 h for complete dissolution of Cs_2CO_3. Afterwards, the temperature was increased to 240 °C under nitrogen and 3 mL of OAM-HCl for $CsPbCl_3$, 3 mL of OAM-HBr for $CsPbBr_3$ or a mixture of 2 mL OAM-HBr and 1 mL OAM-HCl precursor was swiftly injected (OAM-HX precursor solutions were prepared by slowly dissolving 1 mL of hydrochloric acid (HCl (aq.) 37%) and 1.28 mL hydrobromic acid (HBr (aq.) 48% respectively in 10 mL OAM in a 25 mL three neck flask). The heating was removed instantaneously after the injection, and the reaction mixture was cooled to room temperature subsequently using an ice bath. The $CsPbX_3$ NCs were isolated by centrifugation at 10000 rpm for 10 minutes. The precipitate was centrifuged again for 10 minutes at 10000 rpm, while the supernatant was discarded. The remaining supernatant after the second centrifugation was removed with a syringe and the precipitate was collected in 3 mL hexane. After filtering the dissolved NCs through a 0.2 µm PTFE syringe filter, the NCs were stored in a glovebox.

SCs were grown on silicon wafer substrates (10×10 mm^2) according to a two-layer phase diffusion technique under a nitrogen atmosphere. The substrate was placed in a test tube with an inner diameter of 12 mm and covered with 100 µL of a 4 µM stock solution in hexane with additional 500 µL hexane. Afterwards, acetonitrile antisolvent was overlayered with the same amount of the particle solution. Subsequently, the test tube was positioned within a 50 mL centrifuge tube, which was sealed and stored for five days at ambient temperature in dark. Following the crystallization period, the residual solvent was extracted, and the substrate was dried for at least 2 h.

1.3 $CsPbBr_3$ stock solution concentration

A series of dilutions of the $CsPbBr_3$ NC stock solution in hexane was created and the corresponding absorbance spectra recorded. The recorded extinction values at 335 nm and 400 nm, listed in Table S1, were used to calculate the concentration of the stock solution using the molar extinction coefficients ε_λ from Maes *et al.*[2]:

$$\varepsilon_{335\,\mathrm{nm}} = (4.20 \pm 0.04) \cdot 10^{-2} \cdot d^3\ (\mathrm{cm}^{-1} \cdot \mathrm{\mu M}^{-1}),$$

$$\varepsilon_{400\,\mathrm{nm}} = (1.98 \pm 0.04) \cdot 10^{-2} \cdot d^3\ (\mathrm{cm}^{-1} \cdot \mathrm{\mu M}^{-1}),$$

according to eq. S1

$$c_\lambda = \frac{E_\lambda}{\varepsilon_\lambda \cdot f \cdot 1\,\mathrm{cm}}. \qquad (S1)$$

Here $d = 10.8 \pm 0.1$ nm is the average NC edge length in nm (see section 1.4) and f is the stock volume fraction of the dilution.

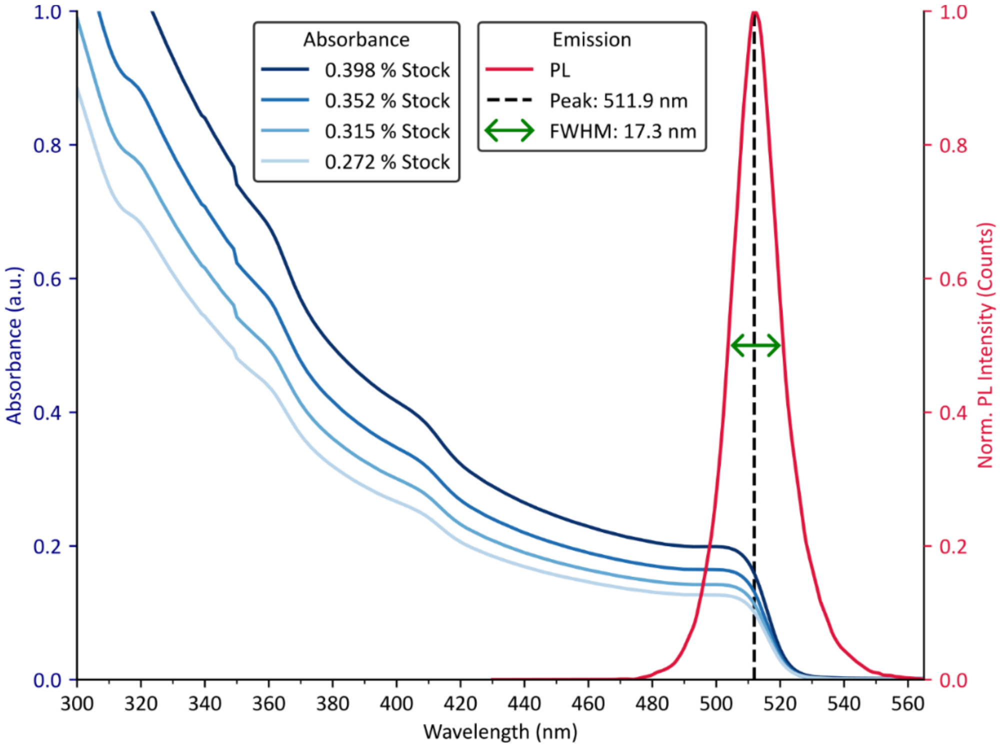

Figure S1. Photoluminescence spectrum and absorbance measurements of a series of dilutions of the $CsPbBr_3$ NC stock solution in hexane. A hexane blank measurement was subtracted from all absorbance measurements. The measurements were performed in a quartz cuvette with a path length of 1 cm. Absorbance spectra were acquired using a UV-VIS-NIR spectrometer (Cary 5000, Agilent Technologies). Emission spectra were acquired on a fluorescence spectrometer (PerkinElmer FL8500).

Table S1. Extinction values E_λ (λ = 335 nm and 400 nm) from absorbance measurements of a concentration series of the $CsPbBr_3$ stock solution displayed in Fig. S1. The concentrations c_λ were calculated according to eq. S1 with the uncertainties resulting from the uncertainties in the molar extinction coefficients.

**Stock Volume Fraction f**	$E_{335\ nm}$	$c_{335\ nm}$ **(µM)**	$E_{400\ nm}$	$c_{400\ nm}$ **(µM)**
0.398 % (6 µl Stock + 1.5 ml Hexane)	0.879826	4.18 ± 0.11	0.416233	4.19 ± 0.14
0.352 % (6 µl Stock + 1.7 ml Hexane)	0.742584	3.99 ± 0.11	0.347051	3.95 ± 0.13
0.315 % (6 µl Stock + 1.9 ml Hexane)	0.647549	3.89 ± 0.11	0.300743	3.83 ± 0.13
0.272 % (6 µl Stock + 2.1 ml Hexane)	0.572480	3.98 ± 0.11	0.266142	3.92 ± 0.13

1.4 $CsPbBr_3$ NC stock solution size distribution

High resolution scanning (transmission) electron micrographs were recorded using a SU8030 (HITACHI) with a resolution of approx. 1.2 nm. Determination of the NC size distribution in the stock solution by scanning transmission electron microscopy is displayed in Fig. S2.

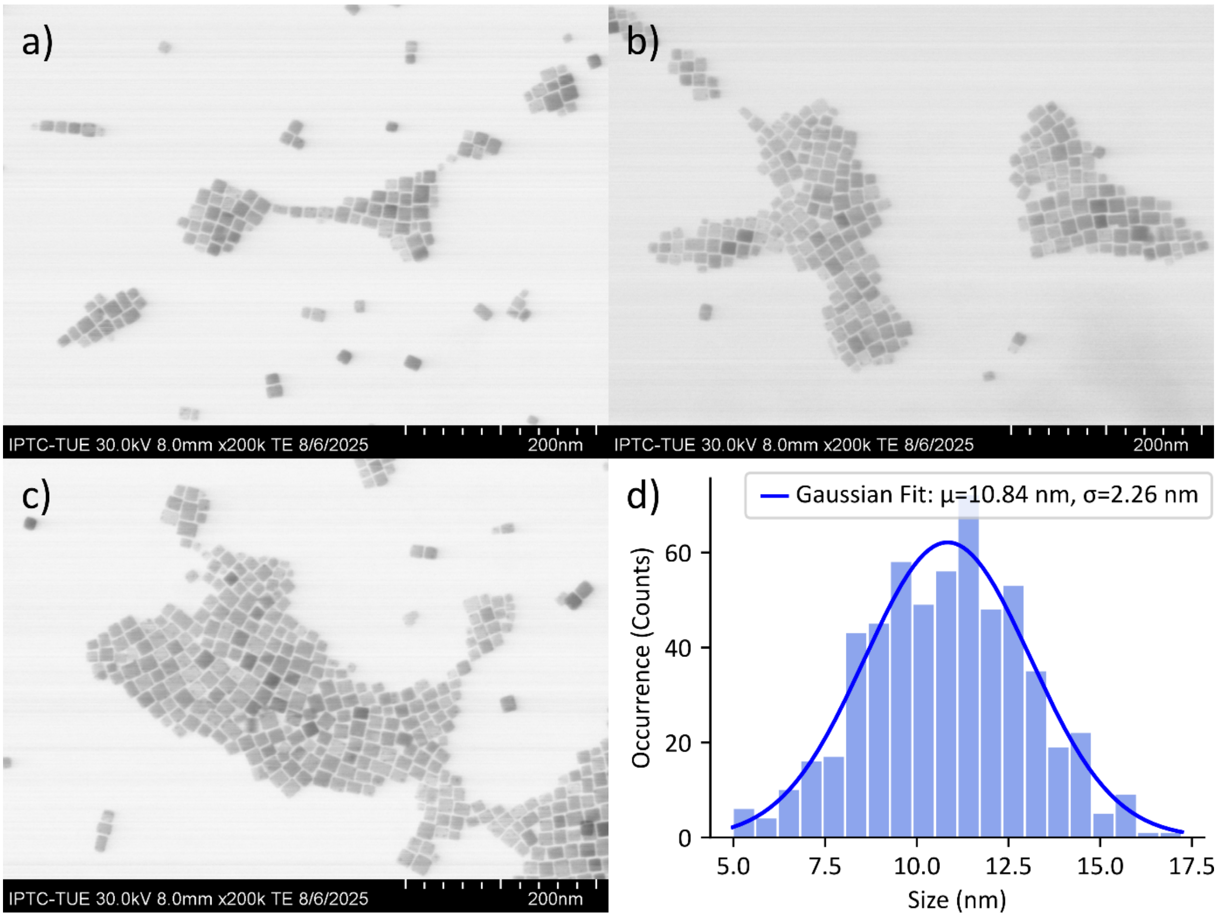

Figure S2. a-c) STEM images of $CsPbBr_3$ NCs dropcasted from the stock solution in hexane on a TEM grid. d) Size distribution of the NCs in the stock solution from measuring the edge lengths of a total number of 571 NCs.

For the NC size determination by STEM, we used the same $CsPbBr_3$ stock solution that has been employed for the assembly of the SCs for the synchrotron experiments. Compared to the NC size distribution values reported in our previous study, which were recorded freshly after the synthesis of the stock solution, we see an increase of the average NC size from 7.5 to 10.8 nm and a broader size distribution.[3] We attribute these changes to aging of the stock solution, as, while stored in a glovebox, nearly two years elapsed between the two measurements.

1.5 Qualitative NMR of oleylamine and oleic acid

^1H NMR spectroscopy was performed at a 400 MHz Bruker Avance HDX NMR spectrometer. For native NC NMR spectroscopy, the NCs were dissolved in toluene-d_8. Quantitative NMR analysis was conducted using 400 µl of 15 mM ethylene carbonate in DMSO-d_6 as an internal standard. All spectra were recorded with the same number of scans, a relaxation time of 40 s, and an acquisition time of 4 s.

In this process, a defined amount of NCs, quantified via UV-VIS spectroscopy, was dissolved in the standard solution, leading to NC decomposition and removal of surface ligands. Quantitative evaluation was based on integration of the internal standard at 4.5 ppm (4.40-4.60 ppm) and the vinyl protons of OA/OAM at 5.3 ppm (5.25-5.4 ppm).

Fig. S3 displays ^{1}H-NMR spectra of the vinyl signals of OAM and OA in the $CsPbBr_3$ NC stock solution. Compared to the sharp vinyl signals of the pure ligands, the signals in the NC stock are broadened and shifted, which is a characteristic signature of surface-bound ligands.[4,5] We do not detect sharp signals corresponding to free OAM or OA in the NC stock solution, indicating that nearly all ligands are bound to NC surfaces.

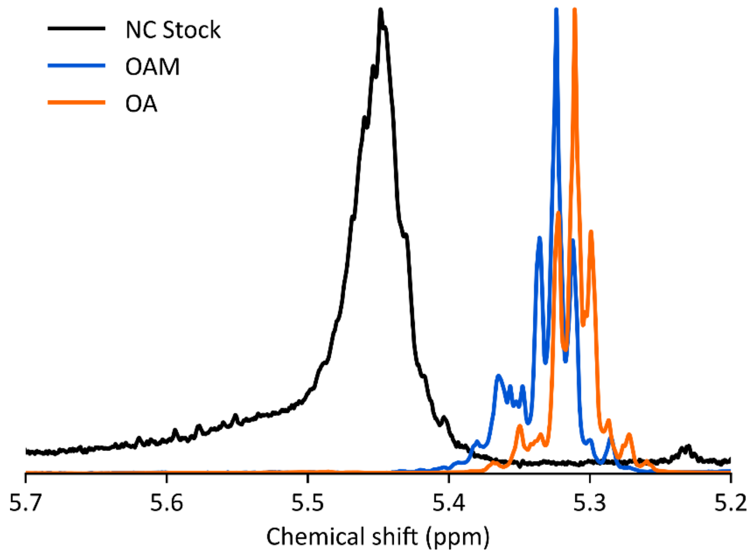

Figure S3. ^{1}H-NMR vinyl signals of the NC stock solution and free oleylamine and oleic acid recorded in toluene-d_8. The signals are normalized and serve as a qualitative reference.

1.6 Quantitative NMR

To quantify the loss of ligands during the two-layer phase diffusion assembly using acetonitrile antisolvent 400 µL of a 15 mM ethylene carbonate in DMSO-d_6 solution was added to all investigated samples to decompose all NCs and release the surface-bound ligands. For the assembly process 600 µL of the diluted NC solution (100 µL of the 4 µM stock solution + 500 µL hexane) were layered on top of 600 µL acetonitrile. Quantitative NMR was performed on four samples: The NC stock solution, from which we obtain the number of ligands/NC prior to the assembly, the residual acetonitrile remaining in the test tube after the assembly, the material adhered to the inner wall of the test tube, and finally the SCs from the crystallization substrate.

Table S2. Quantification of OAM/OA ligands in the samples taken from the two-layer phase diffusion assembly of $CsPbBr_3$ NCs. The corresponding vinyl proton signals are displayed in Fig. 1e of the main text. The coefficient of variation of the integral of the ^{1}H NMR signal of the quantitative standard ($\pm$ 5.4 %) is used as an estimate for the uncertainty associated with the number of ligands recovered from the individual samples.

Sample	Vinyl ^{1}H Integrals I_A	Number of Ligands n_L (mol)	P (%)	P_{norm} (%)
Stock	0.2673	$(8.019 \pm 0.433)\cdot10^{-7}$	100	100
Acetonitrile	0.1554	$(4.662 \pm 0.252)\cdot10^{-7}$	58.14	51
Test Tube	0.1173	$(3.519 \pm 0.190)\cdot10^{-7}$	43.88	38
SC Wafer	0.0328	$(0.984 \pm 0.053)\cdot10^{-7}$	12.27	11

In Table S2, the molar amount of ligands n_L in each sample was calculated from the vinyl proton integrals I_A according to eq. S2

$$n_L = n_{ST} \cdot \frac{I_A}{N_A}, \qquad (S2)$$

where:

n_{ST} = $6 \cdot 10^{-6}$ mol is the molar amount of the external standard (ethylene carbonate),

I_A is the integral of the OA/OAM vinyl proton signals of the sample, and

N_A = 2 is the number of protons contributing to the OA/OAM vinyl proton signals,

P is the percentage of ligands recovered from the individual samples with respect to the number of ligands in the stock solution. P_{norm} represents the same values normalized so that the total ligand recovery equals 100%. The cumulative ligand recovery amounts to 114 % of the ligand content of the stock solution used in the assembly. This slight overestimation reflects the inherent uncertainty of the quantification method, due to pipetting inaccuracies in the amount of employed stock solution or small changes in the NMR integrals of both the vinyl signal and the signal of the quantitative standard.

Several sources of experimental error may contribute to the overall uncertainty. These include measuring and pipetting inaccuracies in the preparation of the DMSO-d_6 standard and the samples as well as instrumental factors related to the NMR measurements itself. In an attempt to quantify the combined effect of these uncertainties, the reproducibility of the integral of the ^{1}H NMR signal of the quantitative standard was evaluated across all four samples: The NC stock solution, the residual acetonitrile remaining in the test tube after the assembly, the material adhered to the inner wall of the test tube, and the SCs from the crystallization substrate. In the absence of experimental errors, this integral should be identical for every spectrum. From integration of the peaks of the internal standard in the range 4.40 to 4.60 ppm, we obtain the mean integral of $9.20 \cdot 10^4$ counts with a standard deviation (1σ) of $4.93 \cdot 10^3$ counts. This corresponds to a coefficient of variation of $\pm$ 5.4 %. In Table S2 we use this coefficient of variation as an estimate for the uncertainty associated with the number of ligands recovered from the individual samples.

The average number of ligands per nanocrystal $n_{L/NC}$ for the different samples is then calculated according to eq. S3:

$$n_{L/NC} = \frac{n_L}{V_{NC} \cdot c_{NC} \cdot f_{NC}}, \qquad (S3)$$

where $V_{NC} = 100\,\mu\mathrm{L}$ is the volume of the stock solution used in the assembly, $c_{NC} = 4\,\mu\mathrm{M}$ is the NC concentration in the stock solution (calculated from the values listed in Table S1), and f_{NC} is the fraction of the NCs added to the assembly that are present in each sample (see below).

For the stock solution, f_{NC} = 1 by definition and $n_{L/NC}$ can be directly calculated. To calculate $n_{L/NC}$ for the crystallization substrate, the f_{NC} value corresponding to the fraction of NCs that deposit on the substrate need to be estimated. The complementary fraction corresponds to NCs adhered to the inner wall of the test tube. Since no NCs were detected in the residual acetonitrile phase, these fractions sum to unity. A photograph of the test tube after the two-layer phase diffusion assembly is depicted in Fig. S4.

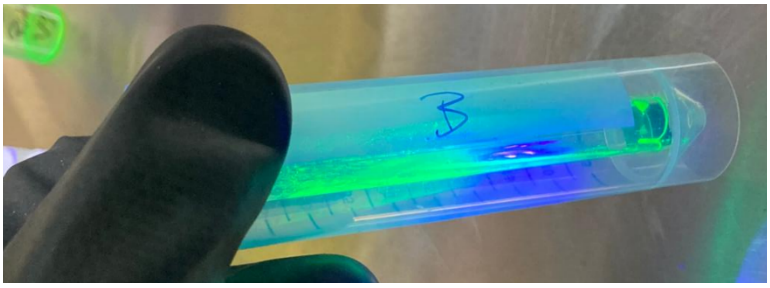

Figure S4. Photograph of the test tube after the two-layer phase diffusion assembly using acetonitrile antisolvent under UV illumination, showing NCs both adhered to the inner wall of the test tube and deposited on the Si substrate at the bottom of the test tube (right side of this rotated image). The residual acetonitrile phase was removed prior to the photograph.

The calculation of the relative substrate coverage is based on optical absorbance spectroscopy of NC dispersions obtained from the crystallization experiments. Five crystallizations were carried out as described in the Materials and Methods section. For each crystallization, NCs were separately extracted from the inner walls of the test tubes and the wafers by redispersing them in 1 mL of hexane. Aliquots of 5, 10, and 15 µL of each dispersion were diluted with 3 mL hexane and absorbance spectra were recorded. From these spectra, the extinction values at 335 nm and 400 nm were determined, and the corresponding concentrations were calculated according to eq. S1 for an average NC size of 8 nm (average size of the NCs in the stock solution used for these experiments). The resulting concentrations, averaged over the three aliquots, are listed in Table S3 together with the relative substrate coverage. This analysis rests on the assumption that the size distribution of the NCs recovered from the crystallization substrate and the test tube are similar.

From our experiments, we estimate that on average 27 ± 6 % of the NC material are deposited on the substrate during crystallization, corresponding to a ligand coverage of 911 ± 210 ligands/NC.

Table S3. Calculated concentrations and distribution of the NCs recovered from the test tubes and the wafers for five different crystallization batches. All concentrations reported here have an associated uncertainty of $\pm$ 1.2 %, resulting from the uncertainties in the molar extinction coefficients employed in their calculation according to eq. S1.

	Batch 1		Batch 2		Batch 3		Batch 4		Batch 5	
	Test tube	*Wafer*	*Test tube*	*Wafer*	*Test tube*	*Wafer*	*Test tube*	*Wafer*	*Test tube*	*Wafer*
$\mathbf{c}$ ($\mu\mathbf{M}$)	0.82	0.21	0.43	0.15	0.43	0.13	0.54	0.24	0.61	0.32
Dist. (%)	80	20	74	26	77	23	69	31	66	34

1.7 Influence of acetonitrile exposure on NC size distribution

Fig. S5 displays STEM measurements detailing the influence of prolonged acetonitrile exposure on the NC size and size distribution. The upper row of Figure R1 shows the size distributions of $CsPbBr_3$ NCs drop-casted from hexane dispersions on TEM grids of the as-synthesized batch (Fig. S5a) and after stirring with the same volume of acetonitrile for approx. 2.5 days (Fig. S5b). The NC size values were determined by measuring the edge lengths in multiple STEM images, examples of which are presented in the lower row.

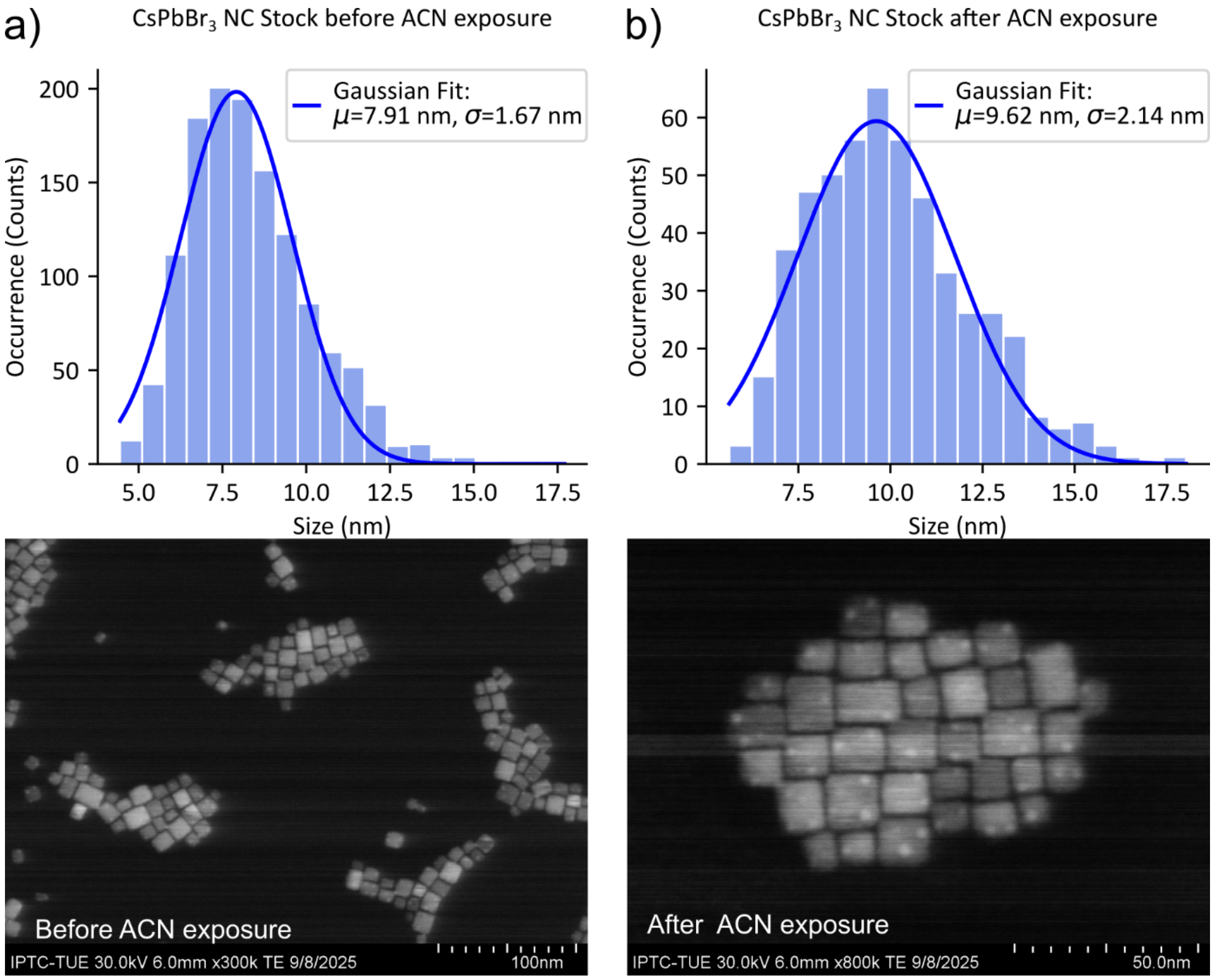

Figure S5. Size distributions (upper) and exemplary STEM images (lower) of $CsPbBr_3$ NCs a) as-synthesized and b) after stirring in acetonitrile for approx. 2.5 days. The NC size histograms result from measuring the edge lengths of a total number of 1275 and 508 NCs, for a) and b) respectively.

The smaller initial average NC size and narrower size distribution of the as-synthesized NC stock solution than in Fig. S2 is due to the use of a different synthesis batch, as the original batch has been depleted. Since this new batch was produced using the same synthesis route, we expect the qualitative effect of acetonitrile exposure to be comparable.

The data shows that acetonitrile exposure induced ligand-stripping goes hand in hand with an increase in the NC size distribution and average NC size, that we attribute to result from NC surface reconstruction process (etching and ripening). This is consistent with Fig. 2e of the main text displaying a varied size distribution in the SC, with containing sizes different than the original standard deviation in Fig. S2.

As In calculating the average number of ligands/NC on our crystallization substrate from our quantitative NMR experiments we don't make assumptions about NC size or size distribution, the NCs found on the crystallization substrates being on average slightly larger than the initial average size employed in the assembly does not alter the reported ligands/NC values.

1.8 Further information on NC self-assembly

The NCs display a pronounced tendency towards self-assembly. When spin-coated from the stock solution in hexane, they typically do not form homogenous NC films but instead organize into small SCs with lateral dimensions of approx. 1x1 μm^2, as displayed in Fig. S6.

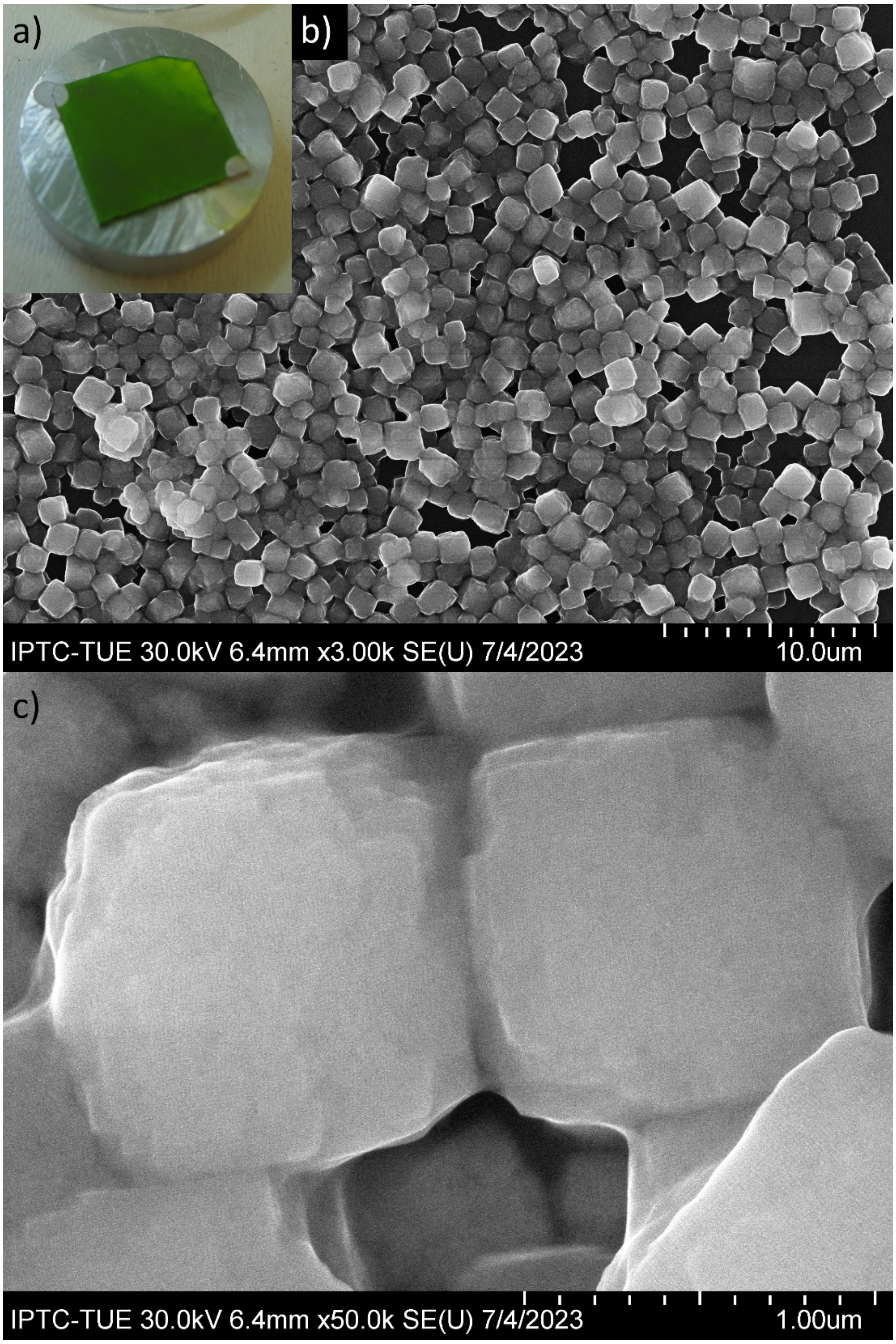

Figure S6. a) Photograph of the Si wafer spin-coated with $CsPbBr_3$ NCs from the stock solution in hexane. b) SEM image of the Si wafer in a), displaying the formation of small SCs. c) High-resolution close-up SEM image, displaying the high degree of order of the constituent NCs in the small SCs.

Compared to solvent evaporation driven self-assembly, the two-layer phase diffusion assembly using acetonitrile antisolvent yields larger and much thicker SCs. Using this assembly, terrace-like height increases are often observed at the very edges of the SCs as shown in Fig. S7.

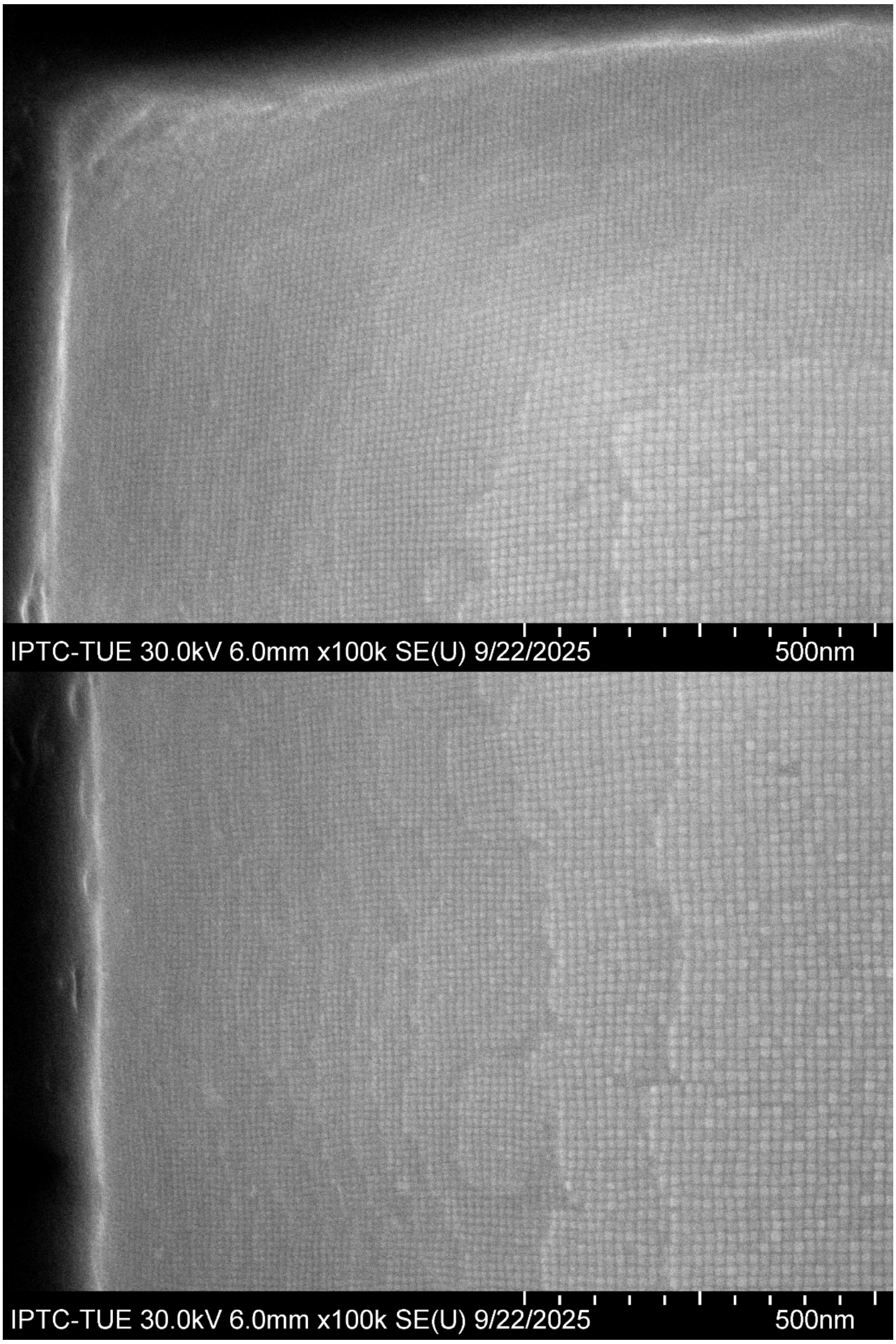

Figure S7. Terrace-like height steps at the edges of $CsPbBr_3$ SCs obtained via the two-layer phase diffusion assembly using acetonitrile antisolvent. The radial NC size gradient with increasingly smaller NCs towards the SC edges is clearly visible.

The SCs obtained from the two-layer phase diffusion assembly using acetonitrile antisolvent are not completely isolated but embedded within a NC film. As displayed in Fig. S8, this film surrounding the SCs is not completely disordered but consists of domains exhibiting high orientational order.

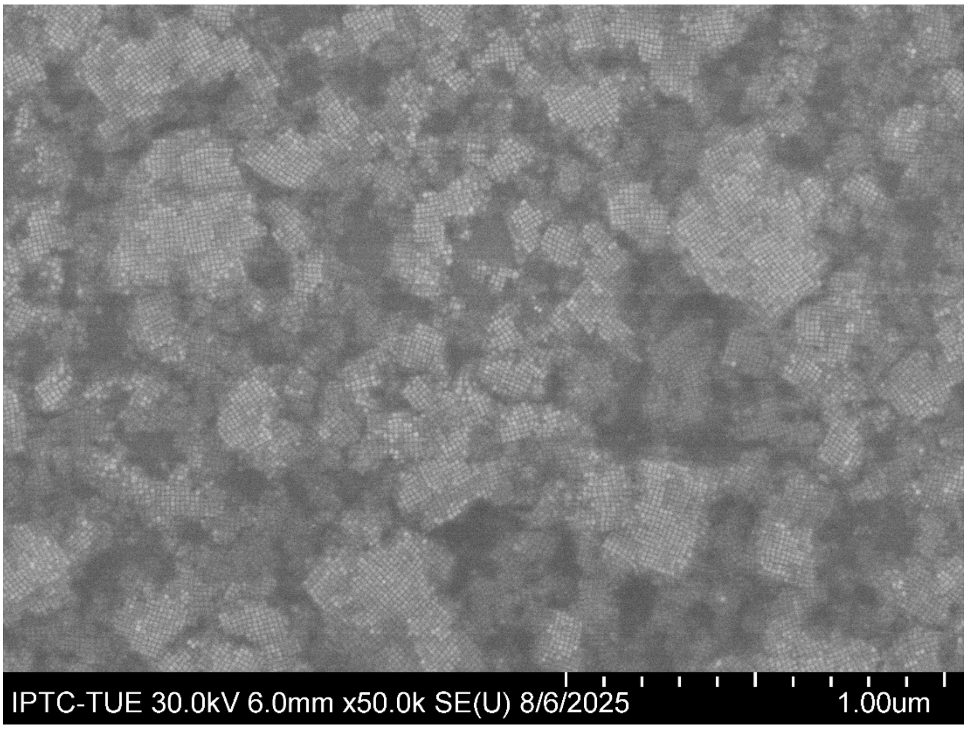

Figure S8. High-resolution SEM image of the film region surrounding the $CsPbBr_3$ SCs obtained from the two-layer phase diffusion assembly using acetonitrile antisolvent.

SCs were transferred using MM3A-EM SEM-compatible micromanipulators (Kleindiek Nanotechnik) equipped with MGS2-EM microgrippers (Kleindiek Nanotechnik) inside a LEO Gemini 1550 VP scanning electron microscope (Zeiss).

2. X-ray nanodiffraction in transmission geometry

A comprehensive description of the general workflow used to evaluate the X-ray nanodiffraction data recorded in transmission geometry can be found in the Supporting Information of our previous work.[3] The following section focuses on addressing the specifics of the WAXS data presented in the present work.

The X-ray nanodiffraction experiments in transmission geometry were conducted at the Coherence Applications beamline P10 of the PETRA III storage ring at DESY using the GINIX endstation.[6,7] Flat-lying and upright standing SCs were measured during different beamtimes with slightly different acquisition parameters.

For the flat-lying SCs the X-ray beam with a photon energy E = 13 keV was focused to approximately $240\ \mathrm{nm\ (vert.)} \times\ 320\ \mathrm{nm\ (hor.)}$. The diffraction patterns were collected by an Eiger X 4M detector placed 398 mm downstream from the sample with an acquisition time of 1 s. Using these acquisition parameters, the SCs in Figs. 2,3,S9, and S10 were raster scanned over a square grid with a step size of approximately 300 nm.

For the upright standing SC the X-ray beam with a photon energy E = 14.6 keV was focused to approximately $500\ \mathrm{nm\ (vert.)} \times\ 430\ \mathrm{nm\ (hor.)}$. Here, the Eiger X 4M detector was placed at a distance of 564 mm from the sample. Using these acquisition parameters, the SC in Fig. 4 was raster scanned over a square grid with a step size of approximately 400 nm.

2.1 Separation of contributions to azimuthal broadening

The measured, total azimuthal peak width (fwhm) $\Delta\phi_m$ contains contributions from orientational disorder $\Delta\phi_{do}$, the finite crystallite size $\Delta\phi_s$, and microstrain $\Delta\phi_\varepsilon$. For the assumption of Gaussian line shapes, these independent broadening mechanisms add in quadrature. The azimuthal peak broadening due to disorder is calculated according to eq. S4,

$$\Delta\phi_{do} = \sqrt{\Delta\phi_m^2 - \Delta\phi_s^2 - \Delta\phi_\varepsilon^2}. \qquad (S4)$$

Finite size broadening is isotropic and contributes equally to both the radial and azimuthal peak width. In reciprocal space, the radial size broadening Δq_s is calculated according to eq. S5,

$$\Delta q_s = \left(\frac{2\pi \cdot K}{s}\right) \qquad (S5)$$

where $K\ =\ 0.85$ is the average shape factor and s is the NC size, obtained from WH analysis for all positions on the SC (Fig. 2e of the main text).

This radial broadening can be translated into azimuthal broadening due to finite NC size $\Delta\phi_s$ according to eq. S6 by considering Δq_s as the arc length on the diffraction ring at q_0

$$\Delta\phi_s = \left(\frac{\Delta q_s}{q_0}\right) = \left(\frac{2\pi \cdot K}{s \cdot q_0}\right). \qquad (S6)$$

The microstrain $\varepsilon = {}^{\Delta a}/_{a}$ obtained from WH analysis for all positions on the SC (Fig. 2e of the main text) is dimensionless and quantifies the distribution of lattice spacings within the nanocrystals. The actual radial peak broadening in reciprocal space due to microstrain is therefore calculated according to eq. S7

$$\Delta q_\varepsilon\ =\ \varepsilon \cdot q_0. \qquad (S7)$$

The associated azimuthal broadening contribution due to microstrain is then calculated according to eq. S8 and is numerically equal to the WH derived microstrain ε itself

$$\Delta\phi_\varepsilon = \left(\frac{\Delta q_\varepsilon}{q_0}\right) = \varepsilon. \qquad (S8)$$

Substituting eqs. S6 and S8 into S4, the azimuthal broadening due to disorder can be calculated directly from maps of $\Delta\phi_m$, s, and ε. Here we note that the contributions $\Delta\phi_s$ and $\Delta\phi_\varepsilon$ constitute only a small correction to the value of $\Delta\phi_m$.

2.2 Calculations of the in-plane atomic lattice parameters

The pseudo-cubic atomic lattice parameter a can be directly calculated from the 100_{AL} reflection according to eq. S9

$$a = \frac{2\pi}{q_0(100_{AL})} \,. \qquad (S9)$$

To calculate the pseudo-cubic atomic lattice parameter b according to eq. S10, the radial positions of both the 100_{AL} and 110_{AL} peaks are required

$$b = \frac{2\pi \cdot a}{\sqrt{a^2 \cdot q_0^2(110_{AL}) - 4\pi^2}}. \qquad (S10)$$

The out-of-plane lattice parameter c cannot be determined from the dataset recorded in transmission geometry as all recorded atomic lattice reflexes have $l = 0$ and therefore do not probe the out of plane spacing. Its calculation from diffraction data in reflection geometry is discussed in Section 3 of the SI.

2.3 Calculation of the average SC lattice parameter

The lattice parameter of the SC $\langle a_{SC} \rangle$ is calculated according to eq. S11, where $\langle q_0 \rangle$ is the average q_0-value of the four first-order SAXS peaks and γ is the angle between the unit vector of the SC unit cell

$$\langle a_{SC} \rangle = \frac{2\pi}{\langle q_0 \rangle \cdot \sin(\gamma)}. \qquad (S11)$$

The angle γ is calculated according to eq. S.12, from the azimuthal peak positions (ϕ_0) of the four first-order SAXS peaks

$$\gamma = \frac{1}{2}\left(\phi_0^{001_{SC}} + \phi_0^{00\bar{1}_{SC}} - \pi\right) - \frac{1}{2}\left(\phi_0^{010_{SC}} + \phi_0^{0\bar{1}0_{SC}} - \pi\right) \,. \qquad (S12)$$

2.4 Instrumental resolution

The magnitude of the scattering vector q is related to the scattering angle 2θ according to eq. S13,

$$q = \frac{4\pi}{\lambda} \cdot \sin(\theta), \qquad (S13)$$

where λ = 0.9537 Å is the X-ray wavelength.

The variation in q with respect to θ is obtained by differentiation and is given in eq. S14

$$\frac{dq}{d\theta} = \frac{4\pi}{\lambda} \cdot \cos(\theta). \qquad (S14)$$

In transmission geometry with a 2D detector at distance L behind the sample, the angle θ can be calculated for any point on the detector at a real-space radial distance R from the direct beam according to eq. S15

$$\theta = \frac{1}{2} \cdot \arctan\left(\frac{R}{L}\right) \qquad (S15)$$

Differentiating eq. S15 with respect to R, we obtain the relation shown in eq. S16

$$\frac{d\theta}{dR} = \frac{L}{2(L^2 + R^2)}. \qquad (S16)$$

Substituting eq. S16 into eq. S14 we can calculate the instrumental resolution in q-space Δq according to the relation in eq. S17, where the pixel size ΔP is the smallest resolvable step in R

$$\Delta q = \frac{4\pi}{\lambda} \cdot \cos(\theta) \cdot \frac{L}{2(L^2 + R^2)} \cdot \Delta P. \qquad (S17)$$

Performing the calculation for L = 400 mm, ΔP = 75 µm, λ = 0.9537 Å (for E = 13 keV) at $q_0(100_{AL})$ = 1.072 Å^{-1}, the obtained value for the instrumental resolution is Δq = 0.0012 Å^{-1}.

As displayed in Fig. S9, the changes in the radial positions of the atomic lattice reflections are discernibly even by eye in the raw diffraction patterns recorded from the edges and the center of the SC.

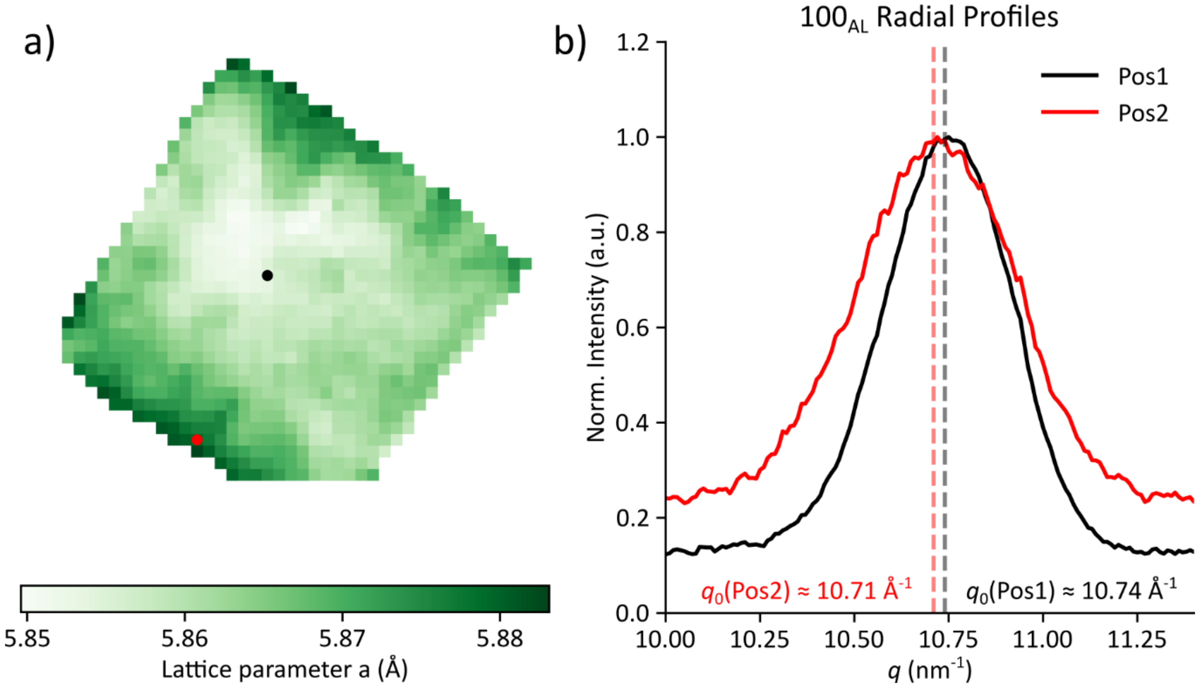

Figure S9. a) Map of the lattice parameter a of the $CsPbBr_3$ SC from Fig.3 of the main text, directly calculated from $q_0(100_{AL})$. b) Raw, unfitted radial profiles of the 100_{AL} reflection recorded from the positions indicated in a) at the center of the SC (Pos1 - black) and at the SC edge (Pos2 - red). The differences in NC size between the center and the edge are evident from the radial peak broadness.

2.5 Spatially-resolved in-plane atomic lattice parameters of a $CsPbBr_2Cl$ and a $CsPbCl_3$ SCs

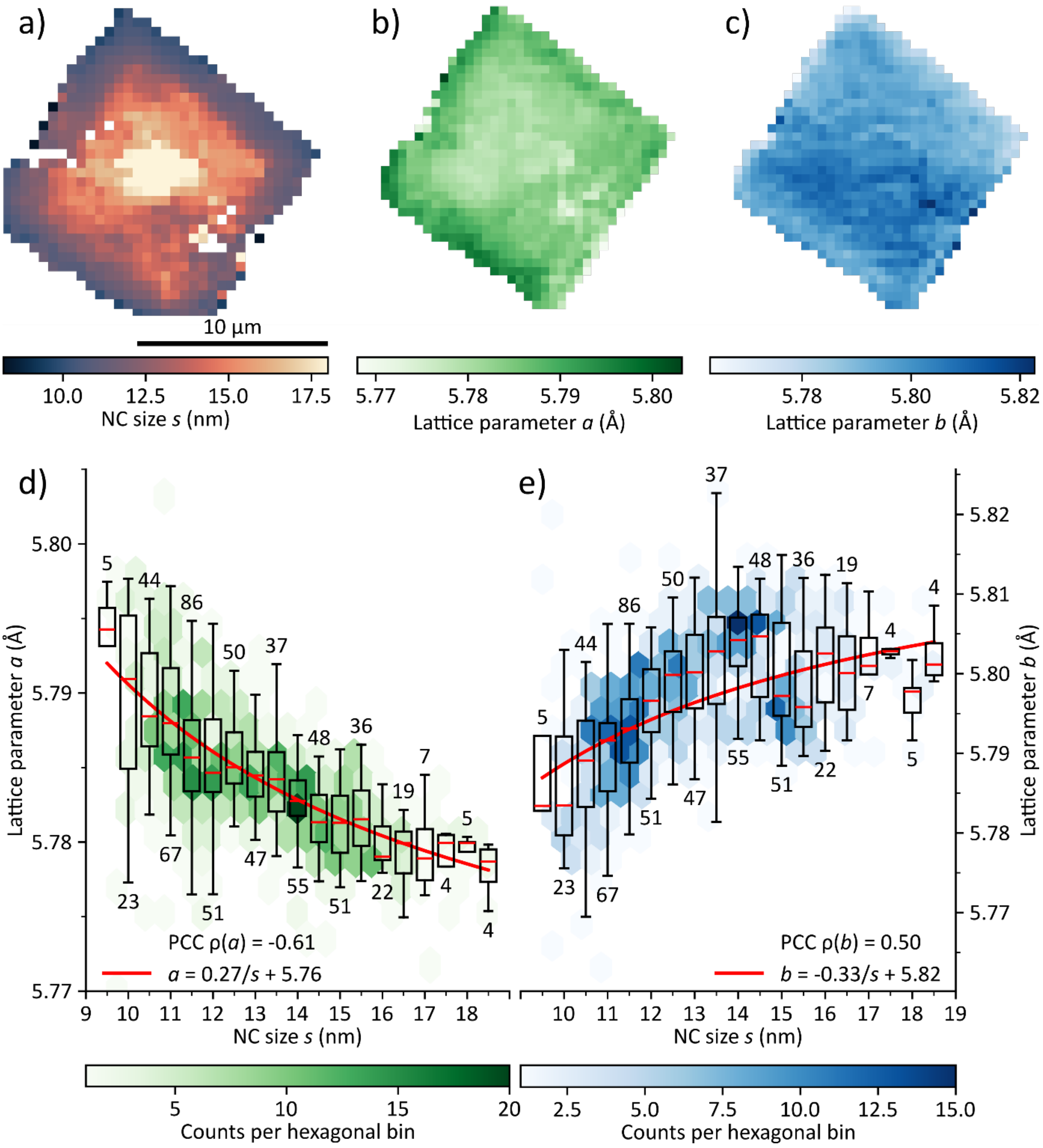

Figure S10. In-plane lattice spacing of a $CsPbBr_2Cl$ SC. a) Map of the NC size s obtained from WH analysis. b) Map of the pseudo-cubic lattice parameter a. b) Map of the pseudo-cubic lattice parameter b. d), e) Correlation of lattice parameters a and b with NC size, respectively. Hexagonal binning displays the statistical distribution, while boxplots indicating the spread and median value within each 0.5 nm size interval. Numbers above the boxplots denote the number of positions contributing to the respective NC size bins. The Pearson correlation coefficients (PCC) ρ are stated to quantify the degree of linear correlation. The median values of the boxplots are fitted using an inverse proportionality with an offset. Missing pixels in a) correspond to diffraction patterns for which WH analysis yielded unphysical results.

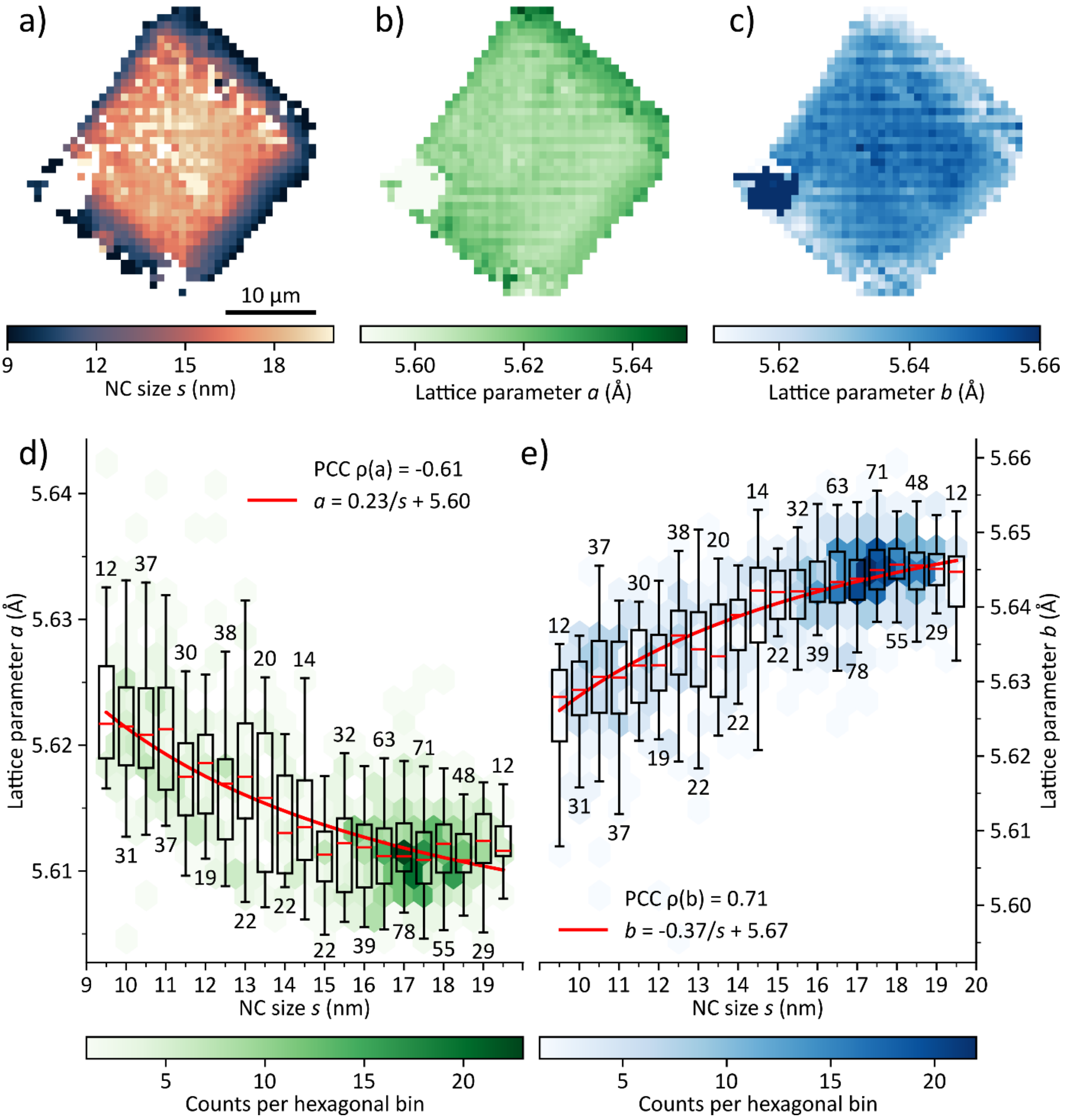

Figure S11. In-plane lattice spacing of a $CsPbCl_3$ SC. a) Map of the NC size s obtained from WH analysis. b) Map of the pseudo-cubic lattice parameter a. b) Map of the pseudo-cubic lattice parameter b. d), e) Correlation of lattice parameters a and b with NC size, respectively. Hexagonal binning displays the statistical distribution, while boxplots indicating the spread and median value within each 0.5 nm size interval. Numbers attached to the boxplots denote the number of positions contributing to the respective NC size bins. The Pearson correlation coefficients (PCC) ρ are stated to quantify the degree of linear correlation. The median values of the boxplots are fitted using an inverse proportionality with an offset. Missing pixels in a) correspond to diffraction patterns for which WH analysis yielded unphysical results.

3. X-ray characterization of as-synthesized $CsPbBr_3$ nanocrystals

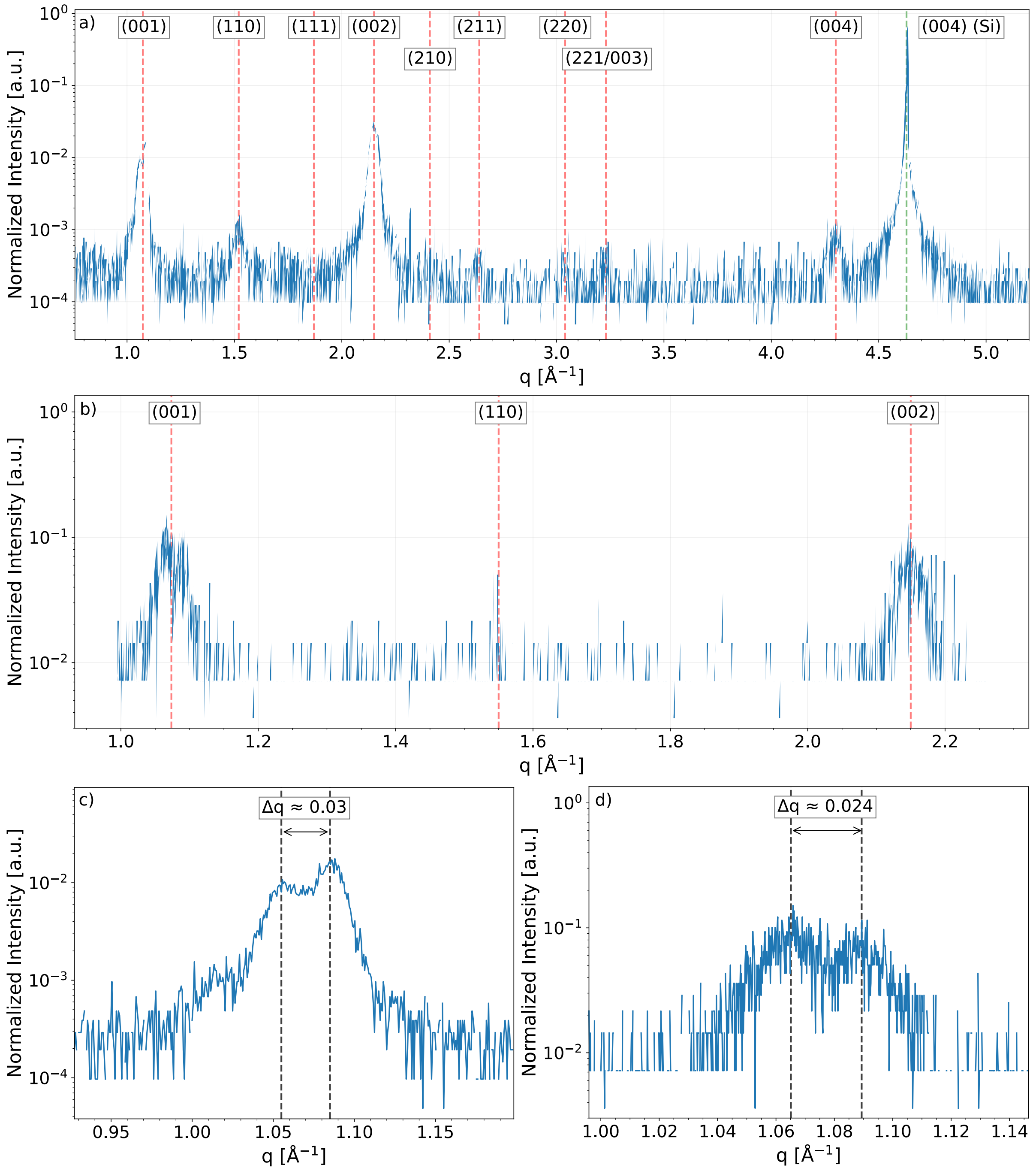

Fig. S12. X-ray diffraction intensity curves of a) dropcast and b) spin coated $CsPbBr_3$ nanocrystals on Si substrates, and c,d) magnified regions around the respective first-order Bragg peaks. Both profiles were measured in $\theta : 2\theta$ reflection geometry. The Bragg peaks of $CsPbBr_3$ are labelled by red dashed lines using cubic notation. The green dashed line denotes the (004) Bragg peak from the silicon substrate. Black dashed lines show the centre of mass for each interference fringe of the split first-order Bragg peak.

We characterized the dropcast and spin-coated $CsPbBr_3$ nanocrystals prior to acetonitrile exposure using the laboratory diffractometer (GE-3303TT, Cu-$K_{\alpha 1}$ radiation) in θ:2θ reflection geometry between the angles $\theta =$ 0-48°, under ambient conditions. Fig. S12a) shows the region $5.5° \leq \theta \leq 39.5°$ of this measurement for a sample prepared via dropcasting and Fig. S12b) shows the region $7° \leq \theta \leq 16°$ for a sample prepared using spin coating techniques. Due to the large beam size, this measurement represents the scattered intensity averaged over all SCs present on the film.

While we acknowledge that there can be difficulties in differentiating between cubic and orthorhombic phases in $CsPbBr_3$ nanocrystals (especially when the signal to noise ratio is high as is found in this measurement), we argue that the measured intensity curve is more consistent with that of a cubic structure, because the position of diffraction peaks align with a cubic structure and no splitting of the peaks is visible, which would indicate deviations of the lattice from a cubic symmetry. The only exception is the split of the (001) peak (see Fig. S12c)-d)). However, we argue that this split originates from the interference of the scattering signal by neighbouring nanocrystals within the superlattice and not from the orthorhombic structure of the nanocrystals.

Previous investigations into the same phenomena show that first-order Bragg peak splitting occurs in a highly ordered cubic NCs as a result of the periodicity of the precisely spaced superlattices within the SC.[8,9] In this case, the X-rays diffracted from the atomic lattice of neighbouring NCs interfere with each other, which results in an apparent splitting of an intrinsically broad first-order Bragg peak from individual NCs. Notably, due to the structural incoherence of the superlattice, there is no splitting expected for the higher order reflections, which is consistent with our data.

Figs. S12c) and d) provide clearer views of the first-order Bragg peak for both preparation methods. We compare the expected peak splitting due to superlattice periodicity to that of the measured value. Assuming an average centre-to-centre distance between NCs of approximately $\Lambda \approx 170$ Å (as shown in Fig. 4d) we can calculate an estimate of the expected peak splitting $\Delta q = 2\pi/\Lambda = 0.03695$ Å^{-1}. This is on the same order of magnitude as the measured value for both dropcast ($\Delta q = 0.03$ Å^{-1}) and spin-coated samples ($\Delta q = 0.024$ Å^{-1}).

Finally, considering the spin-coated sample in particular, previous works[10–12] observe how $CsBrX_3$ nanoparticles crystalize in cubic phase, and notably not orthorhombic phase. They note that this is a stable state even during long exposure to air and ascribe the retention of cubic structure to high-temperature synthesis techniques and surface energy effects stemming from the quantum confinement of nanoparticles. More specifically, the increased band gap energies due to this confinement lead directly to the stabilization of the cubic phase. In conclusion, we are confident that our $CsPbBr_3$ samples show cubic, and not orthorhombic, structure.

4. Additional Spectroscopy Data

4.1 Quantum yields

Quantum yields were measured in solution using an integration sphere mounted in a fluorescence spectrometer (PerkinElmer FL8500).

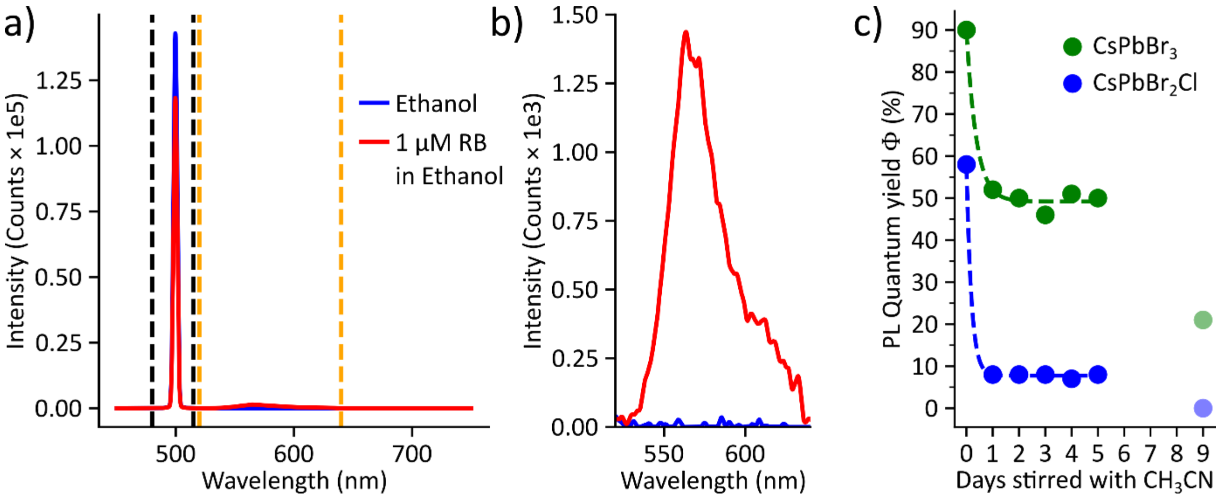

Figure S13. a) Spectra of a 1 µM solution of Rhodamine B in ethanol and the corresponding solvent blank recorded using an integrating sphere as a reference measurement. The excitation (black dashed lines) and emission (orange dashed lines) regions used for integration are indicated. b) Zoom on the emission region. c) Quantum yield measurements performed on $CsPbBr_3$ and $CsPbBr_2Cl$ NCs stirred with acetonitrile antisolvent for different durations.

Fig. S13a) and b) display a reference measurement of a 1 µM solution of Rhodamine B in ethanol excited at 500 nm. As indicated by the dashed lines in Fig. S13a), the excitation and emission regions were defined between 480 – 515 nm and 520 – 640 nm, respectively. The PL quantum yield Φ was calculated according to eq. S18

$$\Phi = \frac{(I_{em}(Sample) - I_{em}(Blank))}{(I_{ex}(Blank) - I_{ex}(Sample))} \cdot 100\%, \qquad (S18)$$

where I_{ex} and I_{em} denote the excitation and emission intensities, integrated over the respective wavelength ranges.

The calculated PL quantum yield of Rhodamine B in ethanol was 64.6 %, in good agreement with literature values for this system.[13]

Fig. S13c) shows PL quantum yields measured for $CsPbBr_3$ and $CsPbBr_2Cl$ NCs stirred with acetonitrile antisolvent as a function of stirring time. Inside a glovebox, vials with stir bars were prepared by overlayering 3 mL of acetonitrile with 40 µL of the NC stock solutions in hexane plus an additional 3 mL of hexane. The vial contents were stirred inside the glovebox to ensure mixing of the two phases. Aliquots were taken approximately every 24 hours for PL quantum yield measurements for the first 5 days. Another data point was acquired after 9 days of stirring. The time point 0 corresponds to the NC stock solutions measured prior to contact with acetonitrile.

A pronounced decrease of the PL quantum yield was observed for both halide compositions within the first 24 hours of stirring with acetonitrile. Specifically, the QY of $CsPbBr_3$ NCs decreased from approx. 90% to around 50%, while $CsPbBr_2Cl$ NCs displayed an even steeper drop of the QY from roughly 60% QY to around 9%. Over the following 5 days, the QY values remained largely constant. However, the dispersions became progressively more turbid, indicating a reduced colloidal stability due to ligand stripping. After 9 days of stirring with acetonitrile $CsPbBr_3$ NCs only displayed around 20 % QY, whereas no photoluminescence could be recorded from the $CsPbBr_2Cl$ NCs.

4.2 TA Spectroscopy

TA spectra were acquired using a spectral broadband femtosecond transient absorption spectrometer from Ultrafast Systems (HELIOS Fire), while a Ti/Sapphire amplifier from Coherent (Astrella-F) was used to generate 100 fs laser pulses at a 1 kHz repetition rate with a central wavelength of 800 nm. The pump pulse being generated by the same source can be tuned to the desired wavelength (here: 475 nm) by an optical parametric amplifier (OPA, Apollo-T). The power was adjusted to low photoexcitation densities of 5 μJ/cm^2. The delay of the probe pulse was tuned by a retro reflector to achieve the desired delay times (up to 7.5 ns). For UV-VIS spectra, the white light continuum (410-750 nm) was created by using a sapphire plate.

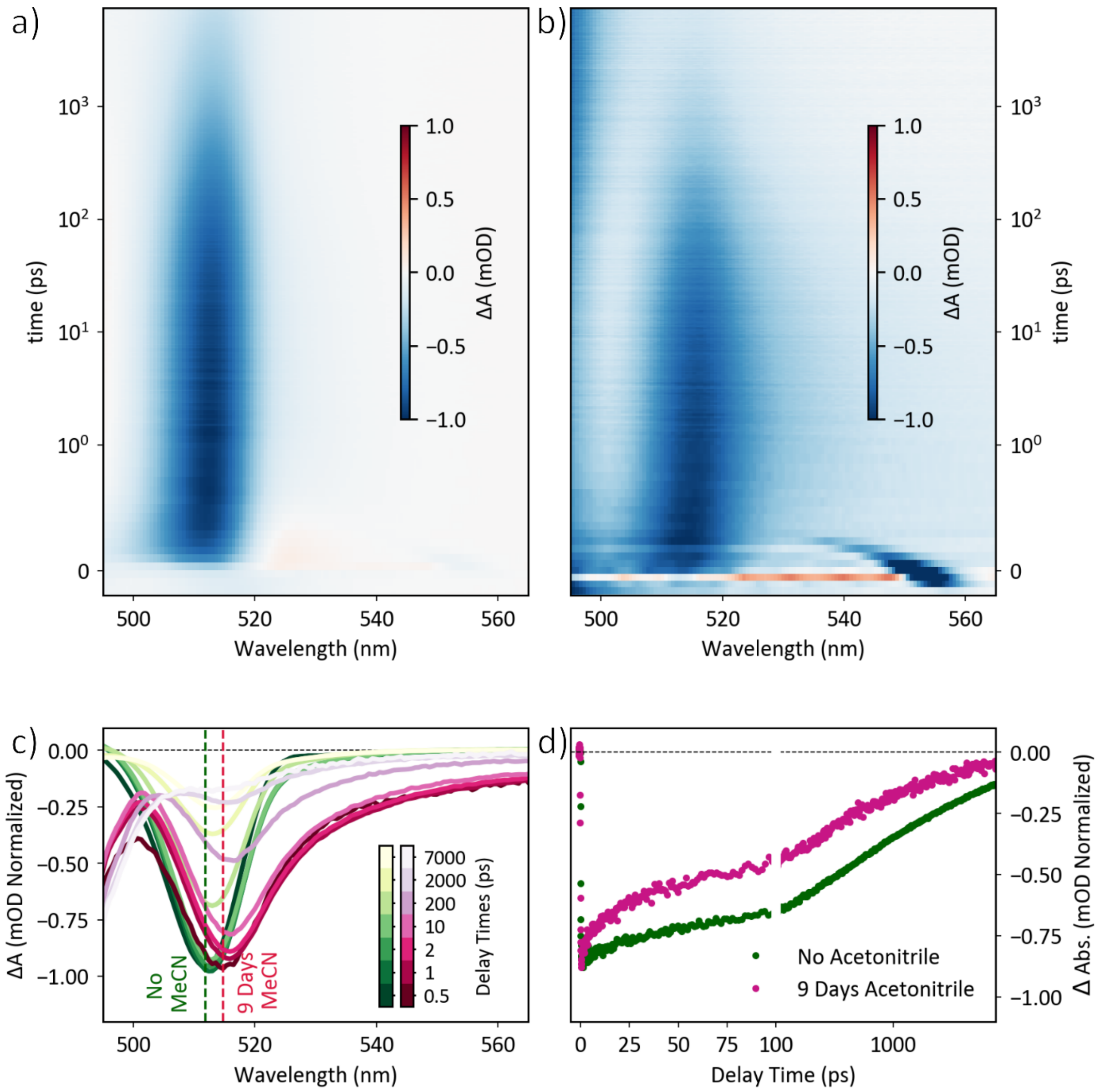

Figure S14: TA hyperspectra of $CsPbBr_3$ NCs dispersed in hexane before (a) and after (b) 9 days exposure to acetonitrile. c) Spectral line cuts of both samples showcasing redshift and broadening. d) Decay traces along the dashed lines in c) showing a faster decay for the exposed sample.

Femtosecond transient absorption (TA) spectroscopy reveals a red-shift of 14 meV, line broadening, and a faster decay of the ground state bleach following acetonitrile exposure, implying that competing nonradiative recombination processes may underly the reduced QY.

4.3 Spatially Resolved PL Spectroscopy

Spectral mapping and TCSPC were performed using a home-built confocal laser piezostage-scanning microscope. A 405 nm laser diode (LDH P-C-405, Picoquant GmbH) emitting picosecond pulses with adjustable repetition rates from 10 to 80 MHz was used as the excitation source. An infinity corrected 100x/0.90 numerical aperture air objective (Carl Zeiss AG) was used to focus the excitation light and collect scattered and emitted light. A 458 nm long-pass filter (RazorEdge LP Edge Filter 458 RU, Semrock) was employed to remove the excitation wavelength from the detection path. Photoluminescence spectra were recorded by a DU4A01-BVF camera (Andor/Oxford Instruments) cooled to -60 °C attached to an SR-303i-B spectrometer (Andor/Oxford Instruments) using a 300 g/mm grating. A single photon avalanche diode (PDM series, Micro Photon Devices) with an active area of 100 µm coupled with time-correlated single photon counting electronics (HydraHarp 400, PicoQuant GmbH) was used to record time-resolved photoluminescence data. Scanning of the piezo stage and interfacing with the spectrometer for spectral mapping was achieved by the HydraLabX1 controller (HydraSpex UG).

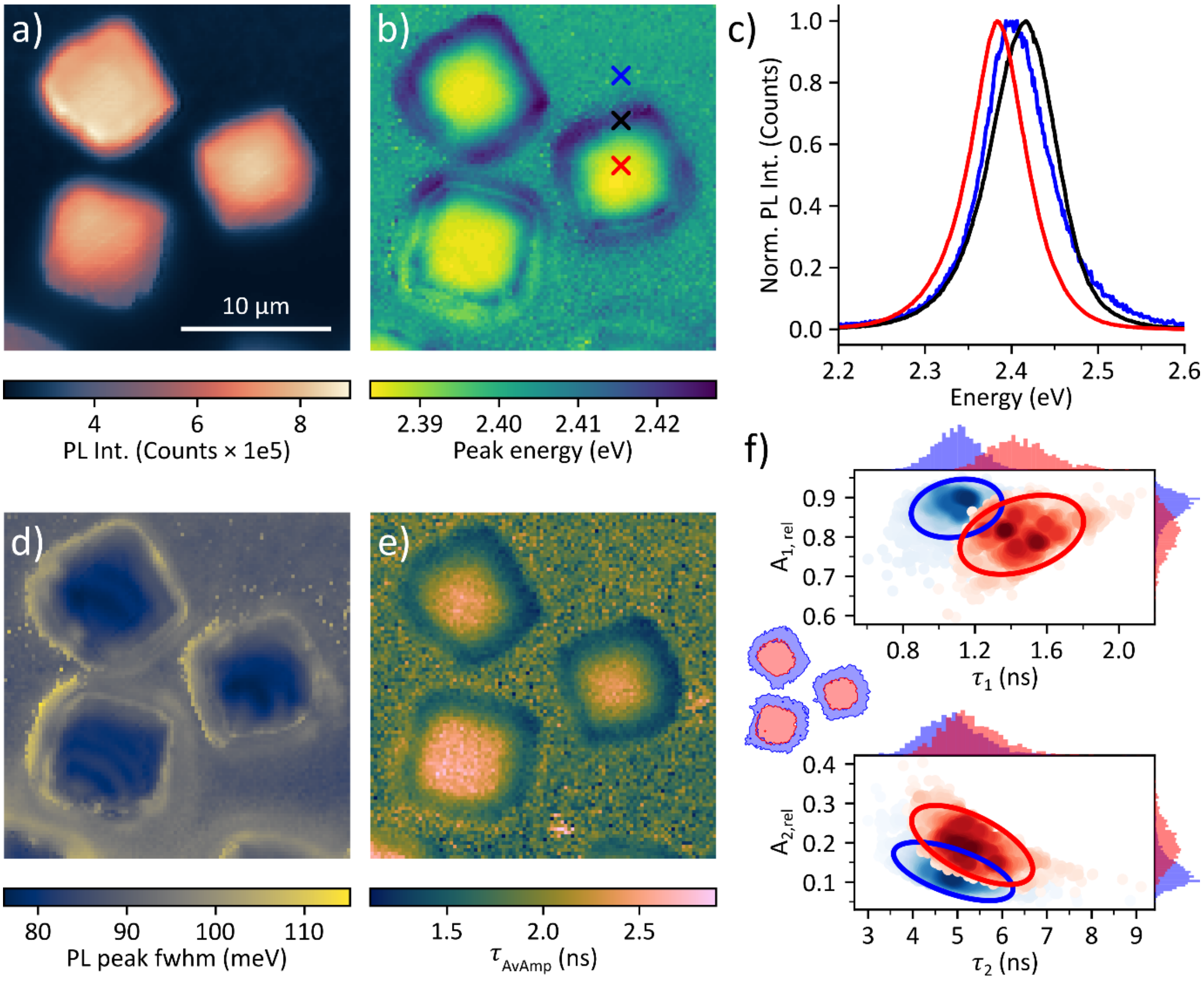

Figure S15. Spatially resolved optical properties of $CsPbBr_3$ SCs obtained from the two-layer phase diffusion assembly using acetonitrile antisolvent. a) Map of the PL intensity. b) Map of the peak emission energy. c) Normalized PL spectra corresponding to the positions marked in b), located outside the SC (blue), at its edge (black), and at the center (red). d) Map of the full width at half maximum (fwhm) of the emission peak. e) Map of the amplitude-weighted average lifetime obtained from biexponential tailfitting of spatially resolved time-correlated single photon counting (TCSPC) data. f) Distributions of the relative amplitudes and lifetimes within the SC extracted from the biexponential fits. Data points are grouped into two regions of interest (ROIs): the inner SC region (red) and the outer SC region (blue). Point density is represented by color. Ellipses indicate 2σ confidence intervals.

Fig. S15a) shows a PL intensity map of three $CsPbBr_3$ SCs embedded in a surrounding film of NCs. The emitted PL intensity from the SCs is approximately three times that of the surrounding film. Within the SCs a slight increase in PL intensity towards the center of each SC is observed. The peak energy map in Fig. S15b) reveals pronounced spatial variations. While the surrounding film of NCs displays a constant emission wavelength of approx. 2.40 eV (516 nm), within the SCs a strong gradient from approx. 2.42 eV (512 nm) at the crystal edges to 2.38 eV (520 nm)

at the crystal center is observed. The SCs are enclosed by a narrow halo region of medium PL intensity (see Fig. 5a) that display distinctly blue-shifted emission compared to both the SC interior and the surrounding film.

Fig. S15c) displays normalized PL spectra from the positions indicated in Fig. S15b): the SC center (red), the SC edge (black), and the surrounding NC film (blue). The peak energies are 2.384 eV at the crystal center (red), 2.400 eV in the NC film (blue), and 2.417 eV at the crystal edge (black). For comparison, $CsPbBr_3$ NCs dispersed in hexane solution emit at 2.422 eV (Fig. S1 - SI). The general redshift due to electronic coupling between the NCs in the assembled superstructures is well documented.[14–16] The red-shifted PL of the surrounding film of approx. 22 meV with respect to the solution, arising from the film displaying partial structural order (Fig. S6), is larger than the value of 10-15 meV, reported by Baranov *et al.* for both dropcast films and pristine isolated SCs.[17] Besides a potential increase in average NC size in the film, we attribute this enhanced red-shift to result from the ligand removal during the assembly facilitating coupling between the nanocrystals.

Within the SCs, the emission shift with respect to the solution is position-dependent. At the SC edges, and in the surrounding halo, the PL maximum coincides with that of NCs in solution. Moving towards the SC center, the emission gradually red-shifts, reaching up to 38 meV. The emission wavelength gradient within the SC can be explained by the radial NC size gradient that is found in SCs assembled using acetonitrile antisolvent. Using the photoluminescence peak-based sizing curve of Brennan et al.,[18,19] the photoluminescence peaks for $CsPbBr_3$ NCs with edge lengths of 10 nm and 15 nm are 511 nm and 519 nm, respectively, which translates to 37 meV energy shift between them, in excellent agreement with 38 meV, suggesting that size segregation is more important than other contributions. At the edges, very small NCs, smaller than the solution average, are incorporated. The blue-shifted emission of these quantum confined NCs and the large orientational disorder at the edges (Fig. 2c) appears to counteract the expected red-shift with respect to the solution PL due to electronic coupling.

Fig. S15d) presents a map of the PL peak fwhm calculated from Voigt profiles fitted to all spectra (Section 4.3 - SI). In the central region of the SCs peak widths of approx. 80 meV are observed, similar to the value recorded for NCs in hexane solution (Fig. S1 - SI). The surrounding NC film is broadened with peak widths on the order of 100 meV. Again, the edges of the SCs deviate strongly from the behavior of the central SC region, displaying broadened emission with fwhms of up to 115 meV. Lineshape analysis reveals a higher Gaussian-to-Lorentzian contribution to the overall PL peak shape in the blue-shifted regions (Figs. S15 and S16 - SI), indicating increased inhomogenous broadening. This is consistent with small NCs with high orientational disorder, which generate a heterogeneous distribution of local emission environments.

Fig. S15e) displays a map of the amplitude-weighted average PL lifetime τ_{AvAmp}, obtained from biexponential tailfitting of spatially resolved TCSPC data. The fitting is displayed in Fig. S16 of the SI. The lowest values of τ_{AvAmp} are recorded at the SC edges, progressively increasing towards the SC centers, with the NC film displaying intermediate values. To quantify the changes in τ_{AvAmp} in the SCs, the extracted biexponential fit parameters (relative amplitudes and lifetimes) from pixels within two regions of interest: the SC interior (red) and the SC edge (blue) are plotted in Fig. S15f). The color-coded ROIs are displayed in the inset. In both regions, the decay is dominated by the fast component ($A_{1,\mathrm{rel}}$ and τ_1). However, the SC interiors show a larger contribution of the slow decay component, together with slightly longer lifetimes for both the short and long component.

4.5 Fluorescence lineshape analysis

Fluorescence lineshape analysis of the spatially-resolved emission spectra presented in Fig. S15 was performed by fitting each spectrum with a Voigt profile as implemented in scipy.special.voigt_profile (SciPy version: 1.15.2, Python version: 3.12.3). This function evaluates the exact convolution of Gaussian and Lorentzian lineshapes through the Faddeeva function.

The extracted σ (the Gaussian standard deviation) and γ (Lorentzian half-width at half-maximum) parameters describe the homogeneous and inhomogeneous broadening contributions, respectively. The Voigt full width at half maximum FWHM_V is approximated using eq. S18, with the Lorentzian FWHM $\mathrm{FWHM}_L = 2\gamma$ and the Gaussian FWHM $\mathrm{FWHM}_G = 2.3548\sigma$.[20]

$$\mathrm{FWHM}_V = 0.5364 \cdot \mathrm{FWHM}_L + \sqrt{0.2166 \cdot \mathrm{FWHM}_L^2 + \mathrm{FWHM}_G^2}. \qquad (S19)$$

To quantify the dominant broadening mechanism, fractional Gaussian and Lorentzian contributions to the overall Voigt profile were estimated by calculating the weighting parameter η according to eq. S20[21]

$$\eta = 1.36603 \cdot \frac{\mathrm{FWHM}_L}{\mathrm{FWHM}_V} - 0.47719 \cdot \left(\frac{\mathrm{FWHM}_L}{\mathrm{FWHM}_V}\right)^2 + 0.11116 \cdot \left(\frac{\mathrm{FWHM}_L}{\mathrm{FWHM}_V}\right)^3. \qquad (S20)$$

Here, $\eta = 0$ corresponds to a purely Gaussian and $\eta = 1$ to a purely Lorentzian lineshape.

Fig. S16c) shows exemplary Voigt fits of the three select positions from Fig. S15. Table S4 displays the extracted parameters.

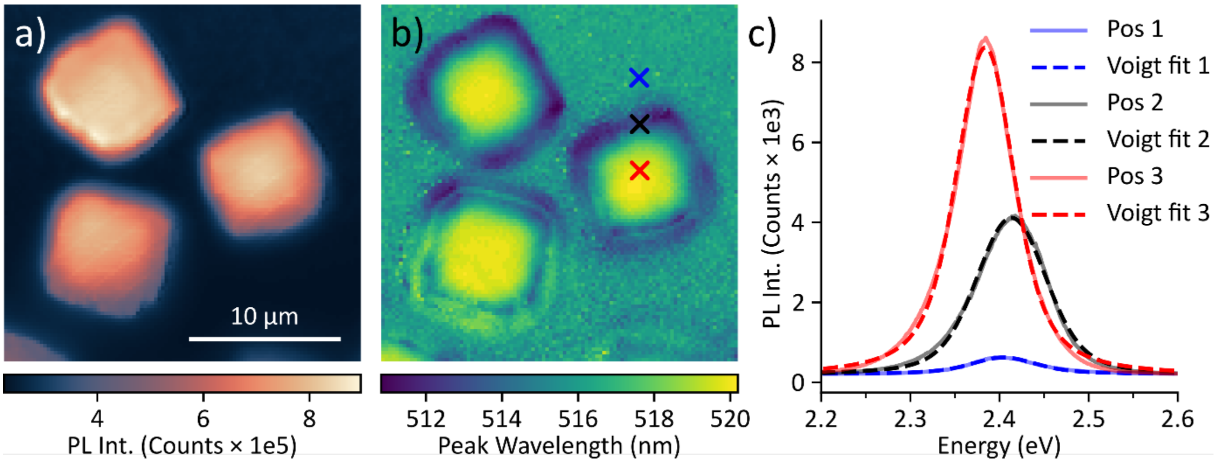

Figure S16. Spatially-resolved optical properties of $CsPbBr_3$ SCs obtained from the two-layer phase diffusion assembly using acetonitrile antisolvent. a) Map of the PL intensity. b) Map of the peak emission wavelength. c) Measured PL spectra and their Voigt fits corresponding to the positions marked in b), located outside the SC (blue), at its edge (black), and at its center (red).

Table S4. Parameters extracted from the Voigt fit of the three spectra in Fig. S16c).

Spectrum	Gaussian Contrib. (%)	Lorentzian Contrib. (%)	σ (eV)	γ (eV)
Pos1 (NC film)	38	62	0.02499	0.02393
Pos2 (SC edge)	60	40	0.03221	0.01541
Pos3 (SC center)	36	64	0.02154	0.02214

The spectra recorded from Pos1 in the NC film and from Pos3 at the SC center are both predominantly Lorentzian in shape. In contrast, the spectrum recorded from Pos2 at the SC edge exhibits a predominantly Gaussian lineshape. To verify whether this is representative of the entire mapped region, lineshape analysis was performed for each spectrum of the spectral map. The resulting parameter maps are displayed in Fig. S17.

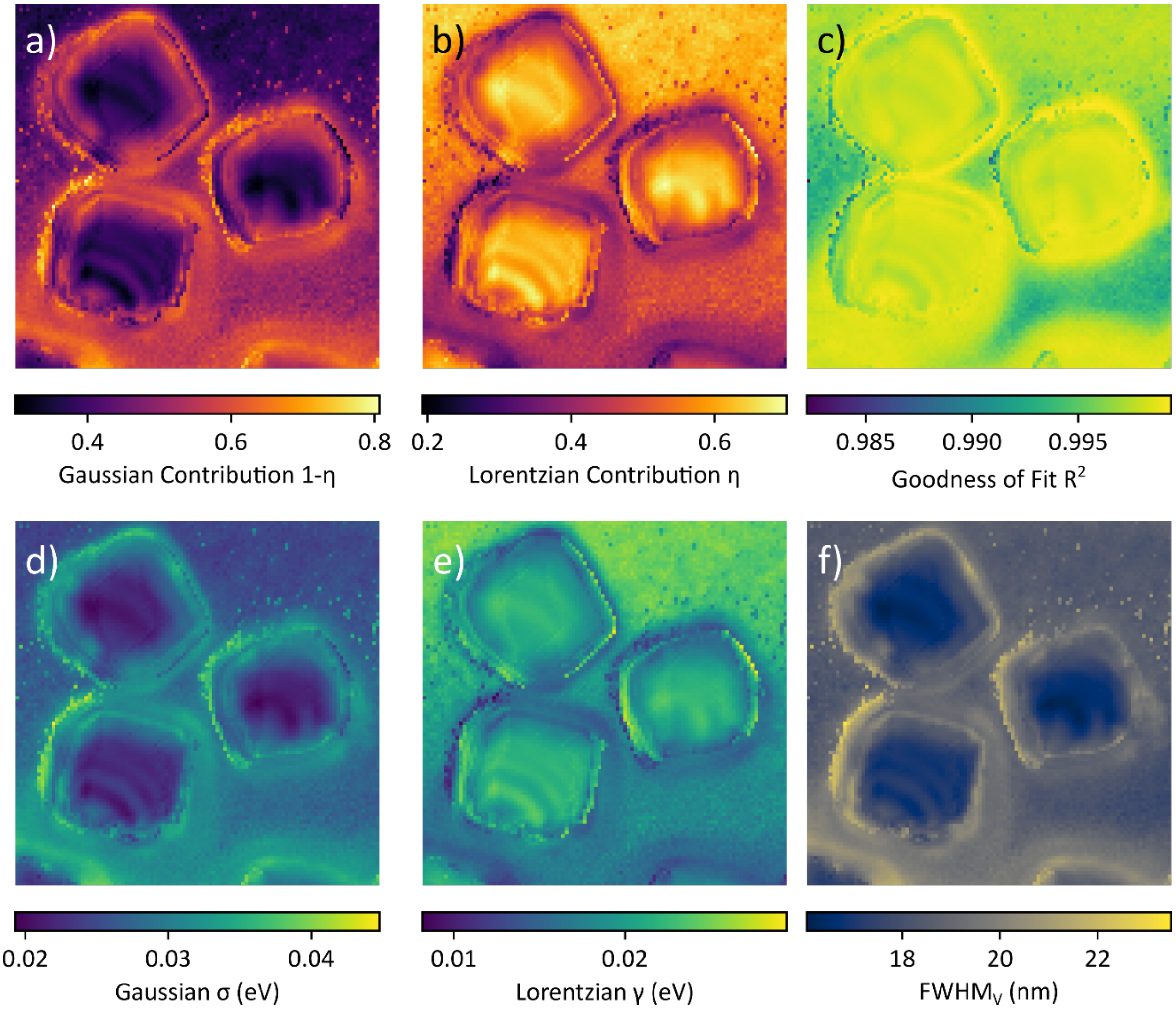

Figure S17. Maps of the PL lineshape parameters extracted from Voigt profiles fitted to all spectra of the spectral map of Fig. S15. a) Map of the Gaussian contribution to the overall Voigt profile. b) Map of the Lorentzian contribution to the overall Voigt profile. c) Map of the goodness of fit. d) Map of the fitted Gaussian standard deviation. e) Map of the fitted Lorentzian half-width at half-maximum. f) Map of the total fwhm of the fitted Voigt profile.

The maps confirm that the SC edges and the surrounding halo region display the highest Gaussian contribution and largest σ values. This is consistent with a pronounced inhomogeneous broadening in these regions displaying blue-shifted emission. The goodness of fit of $R^2 > 0.98$ for all pixels confirms the reliability of the Voigt analysis.

4.6 Time-Correlated Single Photon Counting

Fig. S18a) displays the fitting of the overall PL decay data recorded from a 30x30 µm^2 area of a sample consisting of $CsPbBr_3$ SCs embedded in a thin film of NCs on a Si wafer. The map of the amplitude-weighted lifetime in b) is calculated from the fitting parameters obtained from pixel-by-pixel biexponential tailfitting of the TCSPC data. The upper left region of the lifetime map is displayed in Fig. S15e).

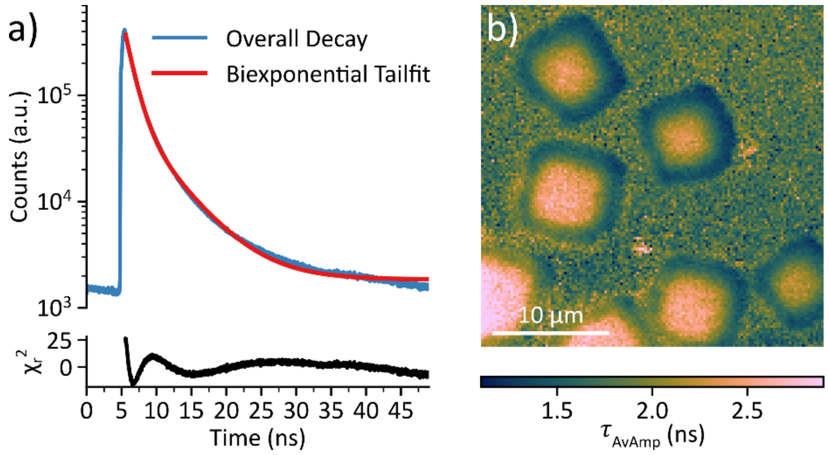

Figure S18. a) Tailfitting of the overall PL decay in the scanned area using a biexponential decay function. The black curve displays the weighted residual χ_r^2. b) Map of the amplitude-weighted average lifetime.

The variability of both the relative amplitudes and the lifetimes of the biexponential decay throughout the image is the reason for the overall decay being only moderately well described by the biexponential model. However, as can be seen in Fig. S19, regions of interest, within which the average lifetime is relatively (but not perfectly) homogenous, are already well described by a biexponential model. Consequentially, the chosen biexponential model is a good fit to the individual histograms recorded from each pixel of the map, which are fitted pixel-by-pixel to generate the lifetime maps displayed in this work. An exemplary single pixel fit is displayed in Fig. S20.

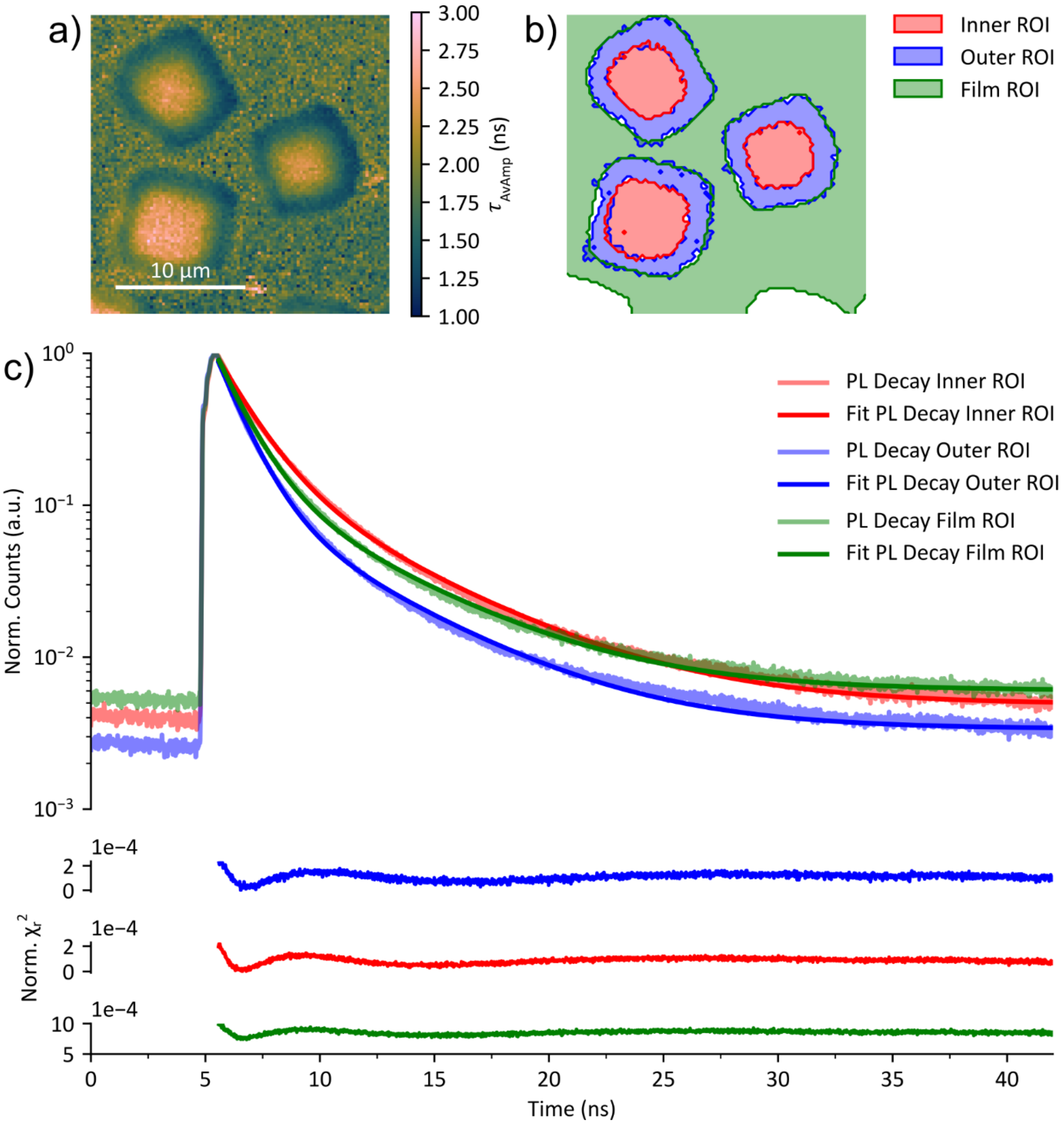

Figure S19. a) Map of the amplitude-weighted average lifetime of the three SCs in the upper left corner of the full lifetime map. b) Three regions of interest within that area within which the recorded lifetimes are relatively homogenous: The inner area of the SCs, the outer area of the SCs, and the surrounding NC film. c) The normalized PL decays of the regions of interest and biexponential tailfits thereof. The curves below display the corresponding normalized weighted residuals of the fits.

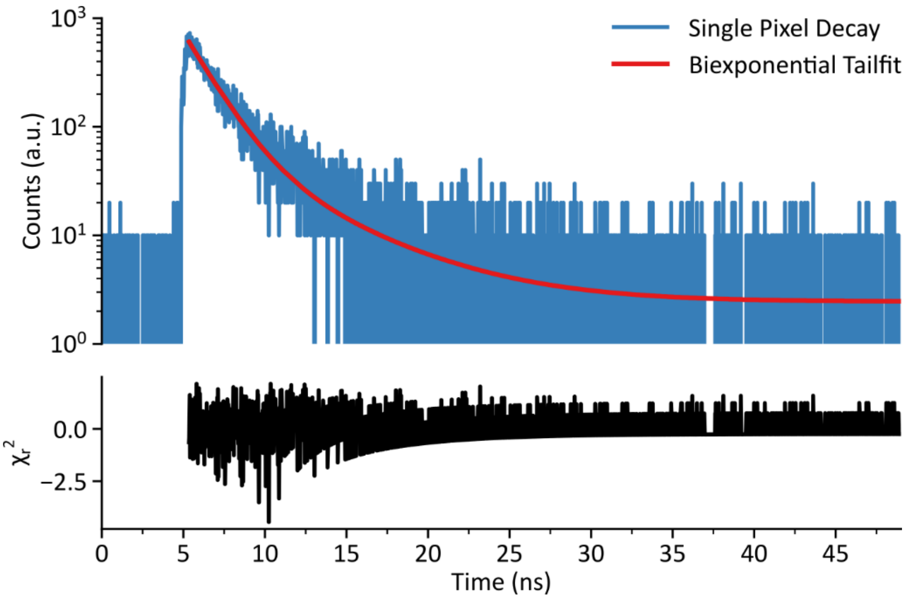

Figure S20. *Biexponential tailfitting of an exemplary single pixel histogram. The black curve displays the weighted residuals.*

References

(1) Dutta, A.; Behera, R. K.; Pal, P.; Baitalik, S.; Pradhan, N. Near-Unity Photoluminescence Quantum Efficiency for All $CsPbX_3$ (X=Cl, Br, and I) Perovskite Nanocrystals: A Generic Synthesis Approach. *Angew. Chem. Int. Ed.* **2019**, *58* (17), 5552–5556. https://doi.org/10.1002/anie.201900374.

(2) Maes, J.; Balcaen, L.; Drijvers, E.; Zhao, Q.; De Roo, J.; Vantomme, A.; Vanhaecke, F.; Geiregat, P.; Hens, Z. Light Absorption Coefficient of $CsPbBr_3$ Perovskite Nanocrystals. *J. Phys. Chem. Lett.* **2018**, *9* (11), 3093–3097. https://doi.org/10.1021/acs.jpclett.8b01065.

(3) Hiller, J. L.; Thalwitzer, R.; Bozkurt, A.; Ferreira, M. G.; Hodak, R.; Strauß, F.; Nadler, E.; Hinsley, G. N.; Wang, B.; Ngoi, K. H.; Rudzinski, W.; Kneschaurek, E.; Roseker, W.; Sprung, M.; Lapkin, D.; Baranov, D.; Schreiber, F.; Vartanyants, I. A.; Scheele, M.; Zaluzhnyy, I. A. Mechanically Robust Supercrystals from Antisolvent-Induced Assembly of Perovskite Nanocrystals. *ACS Nano* **2025**, *19* (28), 26117–26126. https://doi.org/10.1021/acsnano.5c07289.

(4) Ravi, V. K.; Santra, P. K.; Joshi, N.; Chugh, J.; Singh, S. K.; Rensmo, H.; Ghosh, P.; Nag, A. Origin of the Substitution Mechanism for the Binding of Organic Ligands on the Surface of $CsPbBr_3$ Perovskite Nanocubes. *J. Phys. Chem. Lett.* **2017**, *8* (20), 4988–4994. https://doi.org/10.1021/acs.jpclett.7b02192.

(5) De Roo, J.; Ibáñez, M.; Geiregat, P.; Nedelcu, G.; Walravens, W.; Maes, J.; Martins, J. C.; Van Driessche, I.; Kovalenko, M. V.; Hens, Z. Highly Dynamic Ligand Binding and Light Absorption Coefficient of Cesium Lead Bromide Perovskite Nanocrystals. *ACS Nano* **2016**, *10* (2), 2071–2081. https://doi.org/10.1021/acsnano.5b06295.

(6) Kalbfleisch, S.; Neubauer, H.; Krüger, S. P.; Bartels, M.; Osterhoff, M.; Mai, D. D.; Giewekemeyer, K.; Hartmann, B.; Sprung, M.; Salditt, T.; McNulty, I.; Eyberger, C.; Lai, B. The Göttingen Holography Endstation of Beamline P10 at PETRA III⁄DESY; Chicago, Illinois, (USA), 2011; pp 96–99. https://doi.org/10.1063/1.3625313.

(7) Salditt, T.; Osterhoff, M.; Krenkel, M.; Wilke, R. N.; Priebe, M.; Bartels, M.; Kalbfleisch, S.; Sprung, M. Compound Focusing Mirror and X-Ray Waveguide Optics for Coherent Imaging and Nano-Diffraction. *J. Synchrotron Radiat.* **2015**, *22* (4), 867–878. https://doi.org/10.1107/S1600577515007742.

(8) Toso, S.; Baranov, D.; Altamura, D.; Scattarella, F.; Dahl, J.; Wang, X.; Marras, S.; Alivisatos, A. P.; Singer, A.; Giannini, C.; Manna, L. Multilayer Diffraction Reveals That Colloidal Superlattices Approach the Structural Perfection of Single Crystals. *ACS Nano* **2021**, *15* (4), 6243–6256. https://doi.org/10.1021/acsnano.0c08929.

(9) Toso, S.; Baranov, D.; Giannini, C.; Marras, S.; Manna, L. Wide-Angle X-Ray Diffraction Evidence of Structural Coherence in $CsPbBr_3$ Nanocrystal Superlattices. *ACS Mater. Lett.* **2019**, *1* (2), 272–276. https://doi.org/10.1021/acsmaterialslett.9b00217.

(10) Swarnkar, A.; Marshall, A. R.; Sanehira, E. M.; Chernomordik, B. D.; Moore, D. T.; Christians, J. A.; Chakrabarti, T.; Luther, J. M. Quantum Dot–Induced Phase Stabilization of α-$CsPbI_3$ Perovskite for High-Efficiency Photovoltaics. *Science* **2016**, *354* (6308), 92–95. https://doi.org/10.1126/science.aag2700.
(11) Protesescu, L.; Yakunin, S.; Bodnarchuk, M. I.; Krieg, F.; Caputo, R.; Hendon, C. H.; Yang, R. X.; Walsh, A.; Kovalenko, M. V. Nanocrystals of Cesium Lead Halide Perovskites ($CsPbX_3$, X = Cl, Br, and I): Novel Optoelectronic Materials Showing Bright Emission with Wide Color Gamut. *Nano Lett.* **2015**, *15* (6), 3692–3696. https://doi.org/10.1021/nl5048779.
(12) Eperon, G. E.; Paternò, G. M.; Sutton, R. J.; Zampetti, A.; Haghighirad, A. A.; Cacialli, F.; Snaith, H. J. Inorganic Caesium Lead Iodide Perovskite Solar Cells. *J. Mater. Chem. A* **2015**, *3* (39), 19688–19695. https://doi.org/10.1039/C5TA06398A.
(13) Snare, M. J.; Treloar, F. E.; Ghiggino, K. P.; Thistlethwaite, P. J. The Photophysics of Rhodamine B. *J. Photochem.* **1982**, *18* (4), 335–346. https://doi.org/10.1016/0047-2670(82)87023-8.
(14) Tong, Y.; Yao, E.; Manzi, A.; Bladt, E.; Wang, K.; Döblinger, M.; Bals, S.; Müller-Buschbaum, P.; Urban, A. S.; Polavarapu, L.; Feldmann, J. Spontaneous Self-Assembly of Perovskite Nanocrystals into Electronically Coupled Supercrystals: Toward Filling the Green Gap. *Adv. Mater.* **2018**, *30* (29), 1801117. https://doi.org/10.1002/adma.201801117.
(15) Okamoto, T.; Biju, V. Slipping-Free Halide Perovskite Supercrystals from Supramolecularly-Assembled Nanocrystals. *Small* **2023**, *19* (32), 2303496. https://doi.org/10.1002/smll.202303496.
(16) Lazarenkova, O. L.; Balandin, A. A. Miniband Formation in a Quantum Dot Crystal. *J. Appl. Phys.* **2001**, *89* (10), 5509–5515. https://doi.org/10.1063/1.1366662.
(17) Baranov, D.; Toso, S.; Imran, M.; Manna, L. Investigation into the Photoluminescence Red Shift in Cesium Lead Bromide Nanocrystal Superlattices. *J. Phys. Chem. Lett.* **2019**, *10* (3), 655–660. https://doi.org/10.1021/acs.jpclett.9b00178.
(18) Brennan, M. C.; Herr, J. E.; Nguyen-Beck, T. S.; Zinna, J.; Draguta, S.; Rouvimov, S.; Parkhill, J.; Kuno, M. Origin of the Size-Dependent Stokes Shift in $CsPbBr_3$ Perovskite Nanocrystals. *J. Am. Chem. Soc.* **2017**, *139* (35), 12201–12208. https://doi.org/10.1021/jacs.7b05683.
(19) Kuno, M.; Gushchina, I.; Toso, S.; Trepalin, V. No One Size Fits All: Semiconductor Nanocrystal Sizing Curves. *J. Phys. Chem. C* **2022**, *126* (29), 11867–11874. https://doi.org/10.1021/acs.jpcc.2c04734.
(20) Olivero, J. J.; Longbothum, R. L. Empirical Fits to the Voigt Line Width: A Brief Review. *J. Quant. Spectrosc. Radiat. Transf.* **1977**, *17* (2), 233–236. https://doi.org/10.1016/0022-4073(77)90161-3.
(21) Thompson, P.; Cox, D. E.; Hastings, J. B. Rietveld Refinement of Debye–Scherrer Synchrotron X-Ray Data from $Al_2 O_3$. *J. Appl. Crystallogr.* **1987**, *20* (2), 79–83. https://doi.org/10.1107/S0021889887087090.
